# Supplementary material for: Cellular IP6 Levels Limit HIV Production while Viruses that Cannot Efficiently Package IP6 Are Attenuated for Infection and Replication
Source: Cell Rep. 2019 Dec 17;29(12):3983–3996.e4. doi: 10.1016/j.celrep.2019.11.050 (PMC6931105; doi:10.1016/j.celrep.2019.11.050)
Supplement: Document S1. Figures S1–S4 and Table S1 [file mmc1.pdf]

Cell Reports, Volume *29*

Supplemental Information

Cellular IP_6 Levels Limit HIV Production while Viruses that Cannot Efficiently Package IP_6 Are Attenuated for Infection and Replication

Donna L. Mallery, K.M. Rifat Faysal, Alex Kleinpeter, Miranda S.C. Wilson, Marina Vaysburd, Adam J. Fletcher, Mariia Novikova, Till Böcking, Eric O. Freed, Adolfo Saiardi, and Leo C. James

Supplementary Figure 1 related to Figure 1: (A) Analysis of CRISPR knockout clones. Chromatograms and alignments for sequencing of CRISPR/Cas9 knockout clones of IPMK. Indels were identified using the program outlined in Dehairs, J. *et al.*(Dehairs et al., 2016), as well as manual decoding. (B) PAGE and toluidine blue staining of standard cell extracts including standard controls to demonstrate migration of IP_5, IP_6, ATP and GTP. Standards were run in the presence of cell extract to allow equivalent migration. (C) Western blot for Gag expression levels in transfected IPMK KO clones. Gag precursor Pr55Gag (Pr55), p41 and mature capsid protein (p24) are indicated. Lower panel shows loading control COX-IV. (D) Graph showing pg RT per ng p24 in virions from viral supernatants obtained from transfection of IPMK KO clones. Error bars depict mean ± SD of three independent experiments with no statistical difference to WT. (E) Western blots showing overexpression of IPMK in CRISPR knockout clones. Clones were blotted for IPMK and lower panel shows Cox-IV loading control. (F) TiO_2-PAGE and toluidine blue staining of cell extracts from IPMK KO clones stably transduced with empty vector control (EV) or a CMV-driven IPMK gene. Synthetic polyP was used for gel orientation. Gels show knockout clones are successfully reconstituted and IP_6 levels restored. (G) Western blot for Gag expression levels in IPMK KO parental clones and cells stably transduced with either EV or IPMK. Gag precursor Pr55Gag (Pr55), p41 and mature capsid protein (p24) are indicated. Lower panel shows loading control COX-IV.

Supplementary Figure 2 related to Figure 2: (A) Analysis of CRISPR knockout clones. Chromatograms and alignments for sequencing of CRISPR/Cas9 knockout clones of IPPK. Indels were identified using the program outlined in Dehairs, J. *et al.*(Dehairs et al., 2016), as well as manual decoding. (B) Comparison of total IP_5 and IP_6 incorporation in virions from IPPK KO clones shown in Figure 2C. (C) Western blot for Gag expression levels in transfected IPPK KO clones. Gag precursor Pr55Gag (pr55), p41 and mature capsid protein (p24) are indicated. Lower panel shows loading control COX-IV.

Supplementary Figure 3 related to Figure 4: Alignment of lentiviral gag sequences. Residues are highlighted by conservation, with the mature and immature charged ring residues boxed.

Supplementary Figure 4 related to Figure 5: (A) Western blot showing Gag expression levels of lysine mutants in producer cells. Gag precursor Pr55Gag (pr55), p41 and mature capsid

protein (p24) are indicated. (B) Quantification of viral production from 293T, IPMK KO and IPPK KO cells as determined by RT levels in viral supernatants. Error bars depict mean ± SD of two independent experiments. Values are expressed as fold change from levels of RT produced in WT virus. (C) Western blot for Gag expression levels in cell lysates from WT and IPMK and IPPK KO cells during WT and K227I virus production. Gag precursor Pr55Gag (pr55), p41 and mature capsid protein (p24) are indicated. (D) Western blots of cell extracts showing cell extracts from stably transduced cells, showing depletion of TNPO3 or NUP153 by expression of short hairpin RNAs.

Supplementary Table 1 related to Figure 3: Data collection and refinement statistics. Crystallographic statistics for the HIV-1 hexamer structure complexed with IP_5.

Supplementary Figure 1 related to Figure 1

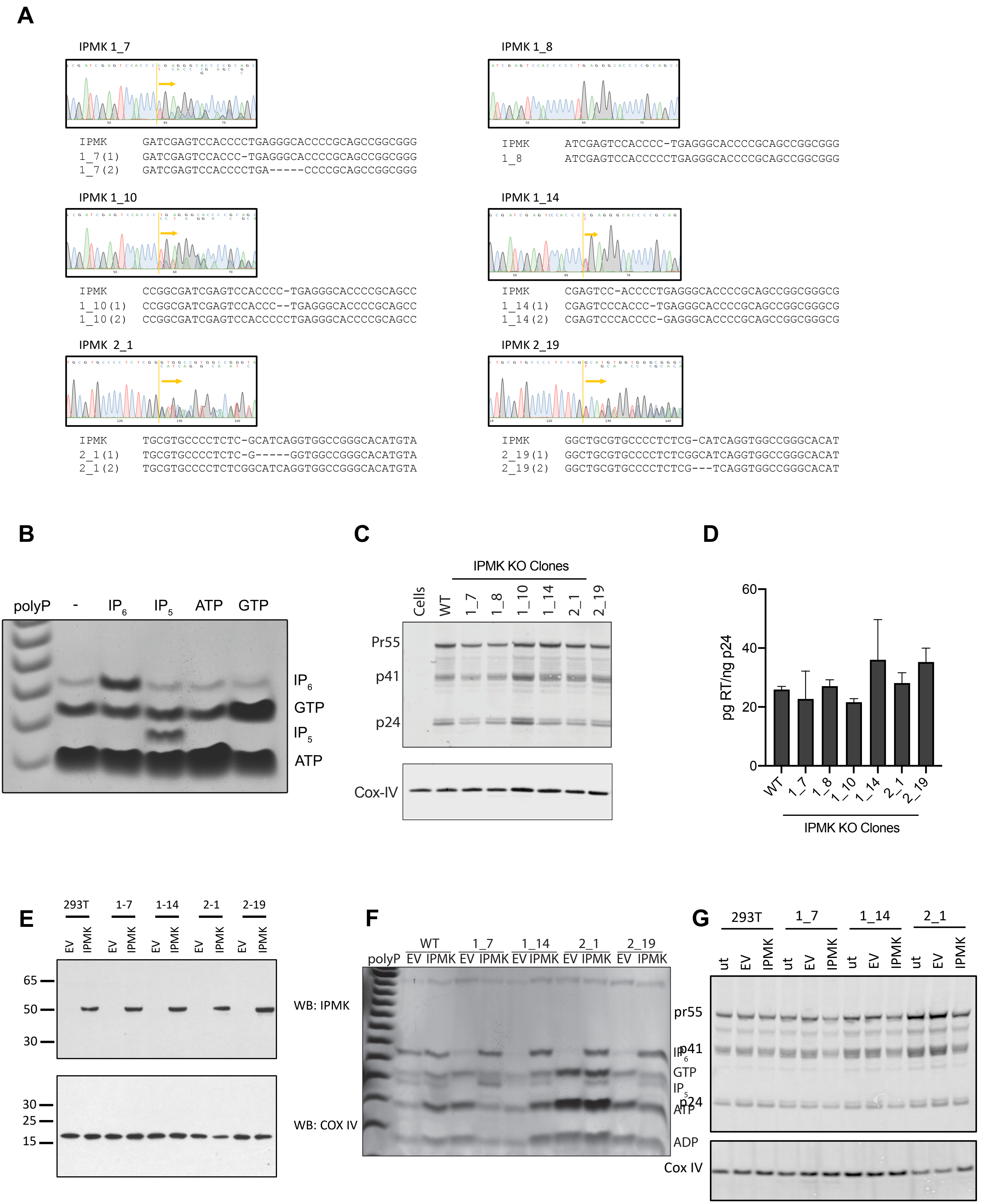

Supplementary Figure 2 related to Figure 2

A

IPPK 1_2

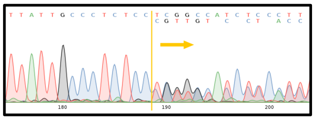

```
IPPK     TATTGCCCTCTCC-GTGGTACCCCCATTCATTCTCGTCC
1_2(1)   TATTGCCCTCTCC---------------------TCGTCC
1_2(2)   TATTGCCCTCTCCCGTGGTACCCCCATTCATTCTCGTCC
```

IPPK B1_9

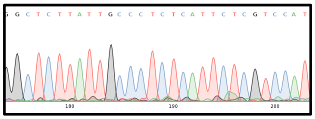

```
IPPK     ATTGCCCTCTCCGTGGTACCCCCATTCATTCTCGTCCAT
1_9      ATTGCCCTCT----------------CATTCTCGTCCAT
```

IPPK B2_2

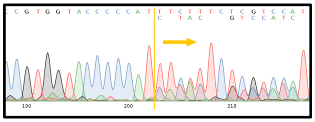

```
IPPK     CGTGGTACCCCCAT-TCATTCTCGTCCATCTTCCCCTCT
2_2(1)   CGTGGTACCCCCATTTCATTCTCGTCCATCTTCCCCTCT
2_2(2)   CGTGGTACCCCCATCT--TTCTCGTCCATCTTCCCCTCT
```

IPPK 2_7

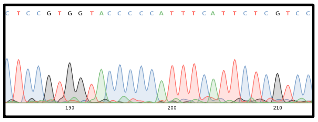

```
IPPK     GCCCTCTCCGTGGTACCCCCA-TTCATTCTCGTCCATCT
2_7      GCCCTCTCCGTGGTACCCCCATTTCATTCTCGTCCATCT
```

IPPK B1_8

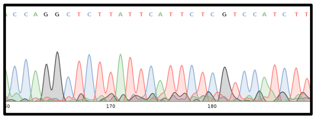

```
IPPK     GCTCTTATTGCCCTCTCCGTGGTACCCCCATTCATTCTCGTCC
1_8      GCTCTTA-----------------------TTCATTCTCGTCC
```

IPPK B1_13

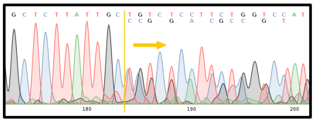

```
IPPK      GCCCTCTCCGTGGTACCCCCATTCATTCTCGTCCATCTTCCCC
1_13(1)   GCCCTCTCC----------------TTCTCGTCCATCTTCCCC
1_13(2)   GCTGGCGCA----------------------------------

IPPK      TCTTCCATGCCCAGGCGCAGCCCTGGCGCCTCGCGGGCTAGGA
1_13(1)   TCTTCCATGCCCAGGCGCAGCCCTGGCGCCTCGCGGGCTAGGA
1_13(2)   ------------------------------TCGCGGGCTAGGA
```

IPPK B2_3

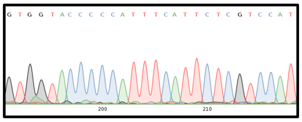

```
IPPK     CTCTCCGTGGTACCCCCA-TTCATTCTCGTCCATCTTCC
2_3      CTCTCCGTGGTACCCCCATTTCATTCTCGTCCATCTTCC
```

IPPK B3_7

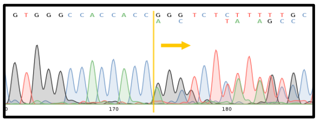

```
IPPK     CGCGTGGGCCACCACCAGG-CTCTTATTGCCCTCTCCGT
3_7(1)   CGCGTGGGCCACCACC--GGCTCTTATTGCCCTCTCCGT
3_7(2)   CGCGTGGGCCACCACCAGGTCTCTTATTGCCCTCTCCGT
```

B

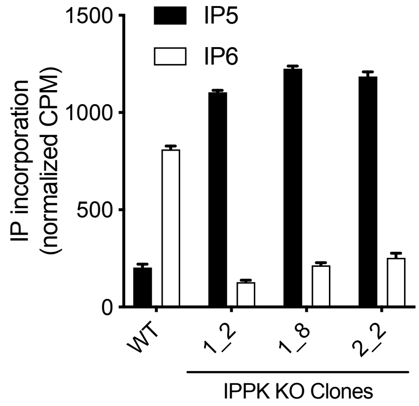

C

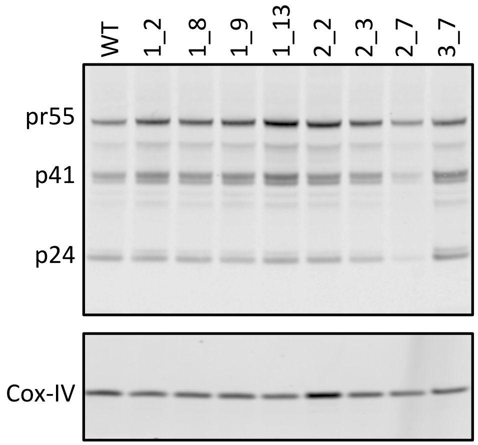

Supplementary Figure 3 related to Figure 4

```
HIV-1_M   1 MGARASVLSGGELDRWEKIRLRPGGK-KKYKLKHIVWASRELERFAVNPG--LLETSEGCRQILGQLQPSLQ--TGSEELRSLYN  80
HIV-1_O   1 MGARASVLTGSKLDAWEQIRLKPGCK-KKYRLKHLVWASRELDRFACNPE--LLETAEGNEQLLQQLEPALK--TGSESLQSLWN  80
HIV-2     1 MGARNSVLRGKKADELERIRLRPGGK-KKYRLKHIVWAANKLDRFGLAES--LLESKEGCQKILTVLDPMVP--TGSENLKSLFN  80
SIVmac    1 MGARNAVLSGKKADELEKIRLRPGGK-KKYMLKHVVWAANELDRFGLAES--LLENKEGCQKILSVLAPLVP--TGSENLKSLYN  80
FIV       1 MGNGQGRDWKMAIKRCSNVAVGVGGKSKKFGEGNFRWAIRMANVSTGREPGDIPETLDQLRLVICDLQERREKFGSSKEIDMAIV  85

HIV-1_M  81 TVATLYCVHQRIEIKDTKEALDKIEEEQNKSKKKAQQAADTG---HSSQVSQNYPIVQNIQGQMVHQAISPRTLNAWVKVVEE- 161
HIV-1_O  81 TIAVLWCVHNRFRVEDTQQAIQKLKEVMG-SRKSAGAAKEDT----SARQTGQNYPVVANAQGQMVHQSLSPRTLNAWVKAVEE- 159
HIV-2    81 TVCVIWCIHAEEKVKDTEGAKQIVRRHLVAETGTAEKMPSTSRPTAPSSEKGGNYP-VQHVGGNYTHIPLSPRTLNAWVKLVEE- 163
SIVmac   81 TVCVIWCIHAEEKVKHTEEAKQIVQRHLVVETGTAETMPKTSRPTAPSSGRGGNYP-VQQIGGNYVHLPLSPRTLNAWVKLIEE- 163
FIV      86 TLKVFAVAGLLNMTVSTAAAAENMYSQMGLDTRPSMKEAGGK-----EEGPPQAYP-IQTVNGVPQYVALDPKMVSIFMEKARE- 163

HIV-1_M 162 KAFSPEVIPMFSALSEGATPQDLNTMLNTVGGHQAAMQMLKETINEEAAEWDRVHPVHAGPIAPGQMREPRGSD--IAGTTSTLQ 244
HIV-1_O 160 KAFNPEIIPMFMALSEGAIPYDTNTMLNAIGGHQGALQVLKEVINEEAAEWDRTHPPPIGPLPPGQIREPTGSD--IAGTTSTQQ 242
HIV-2   164 KKFGAEVVPGFQALSEGCTPYDINQMLNCVGDHQAAMQIIREIINEEAAEWDVQHP-IPGPLPAGQLREPRGSD--IAGTTSTVE 245
SIVmac  164 KKFGAEVVPGFQALSEGCTPYDINQMLNCVGDHQAAMQIIRDIINEEAADWDLQHP-QPAPQ-QGQLREPSGSD--IAGTTSSVD 244
FIV     164 GLGGEEVQLWFTAFSANLTPTDMATLIMAAPGCAADKEILDESLKQLTAEYDRTHP----PDAPRPLPYFTAAE--IMGIGLTQE 242

HIV-1_M 245 EQIGWMTN-NPPIPVGEIYKRWIILGLNKIVRMYSPTS-ILDIRQGPKEPFRDYVDRFYKTLRAEQASQEVKNWMTETLLVQNAN 327
HIV-1_O 243 EQVHWTTRPNNPIPVGDIYRKWIVLGLNKVVKMYSPVS-ILDIRQGPKEPFRDYVDRFYKTLRAEQATQEVKNWMTETLLVQNAN 326
HIV-2   246 EQIQWMFRPQNPVPVGNIYRRWIQIGLQKCVRMYNPTN-ILDIKQGPKEPFQSYVDRFYKSLRAEQTDPAVKNWMTQTLLVQNAN 329
SIVmac  245 EQIQWMYRQQNPIPVGNIYRRWIQLRLQKCVRMYNPIN-ILDVKQRPKEPFQSYVDRFYKSLRAEQTDAAVKNWMTQTLLIQNAN 328
FIV     243 QQAEARFA-----PARMQCRAWYLEALGKLAAIKAKSPRAVQLRQGAKEDYSSFIDRLFAQIDQEQNTAEVKLYLKQSLSIANAN 322

HIV-1_M 328 PDCKTILKALGPAATLEEMMTACQGVGGPGHKARVLAEAMSQVTNT----ATIMMQRGNFRNQRKMVKCFNCGKEGHTARNCRAP 408
HIV-1_O 327 PDCKQILKSLGPGATLEEMMIACQGVGGPTHKARVLAEAMAAAQDLKGGYTAVFMQRGQNPSRKGPIKCFNCGKEGHLARNCRAP 411
HIV-2   330 PDCKLVLKGLGMNPTLEEMLTACQGVGGPGQKARLMAEALKEVIGP----APIPFAAAQQ---RKAFKCWNCGKEGHSARQCRAP 407
SIVmac  329 PDCKLVLKGLGVNPTLEEMLTACQGVGGPGQKARLMAEALKEALRP----VPTPFAAAQQRGPRKPIKCWNCGKEGHSARQCRAP 409
FIV     323 ADCKKAMSHLKPESTLEEKLRACQEIGSPGYKMQLLAEALTKVQVVQ--------------SKGSGPVCFNCKKPGHLARQCREV 393

HIV-1_M 409 RKKGCWKCGKEGHQMKDCT-ERQANFLGKIWPSYKGRPGNFLQSRPEPTAPPFLQSRPEPTAPPEESFRSGVETTTPPQKQEPID 492
HIV-1_O 412 RKKGCWKCGQEGHQMKDCRNGKQANFLGKYWPPGGTRPGNYAQRQVSPSAPPMTEEMK------------GQEN----QEQKEDQ 480
HIV-2   408 RRQGCWKCGKPGHIMTNCP-DRQAGFLG--LGPWGKKPRNFPVAQVPQGLTPTAPPVDPAVDLLEKYMQQGKRQRE--QRERPYK 487
SIVmac  410 RRQRCWKCGKMDHVMAKCP-DRQAGFLG--LGPWGKKPRNFPMAQVHQGLTPTAPPEDPAVDLLKNYMQLGKQQRE--SREKPYK 489
FIV     394 KK--CNKCGKPGHLAAKCWQGNRKNSGN-----WKAGRAAPVNQMQQAVMPSAPPME------------------------- 444

HIV-1_M 493 KELYPLTSL------------------RSLFGNDPSSQ 512
HIV-1_O 481 NELYPFASL------------------KSLFGTDQ--- 497
HIV-2   488 EVTEDLLHLEQGETPYREPPTEDLLHLNSLFGKD---- 521
SIVmac  490 EVTEDLLHL------------------NSLFGGDQ--- 506
FIV     445 ---EKLLDL----------------------------- 450
```

Supplementary Figure 4 related to Figure 5

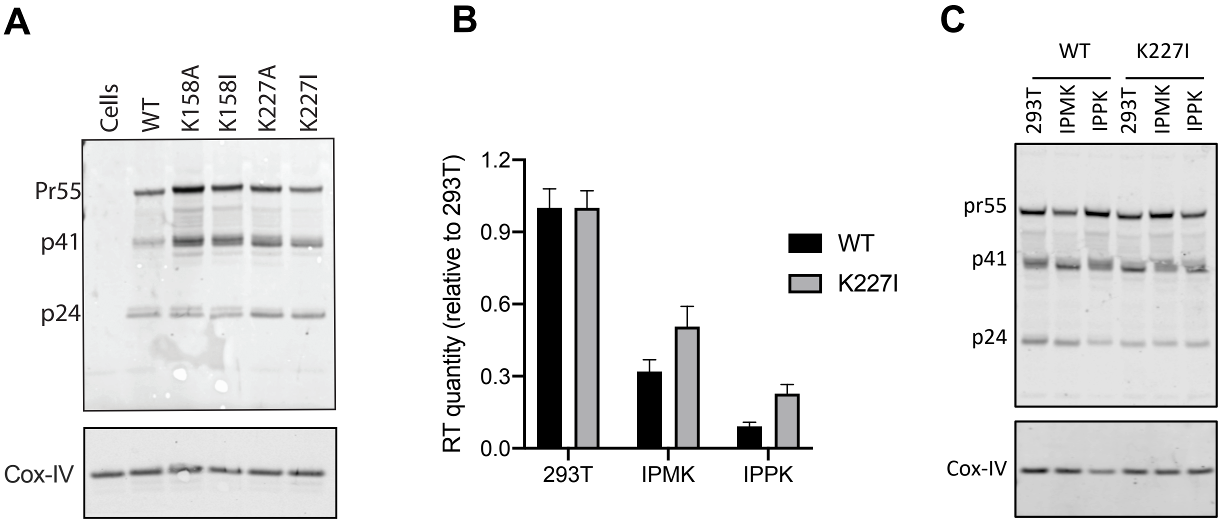

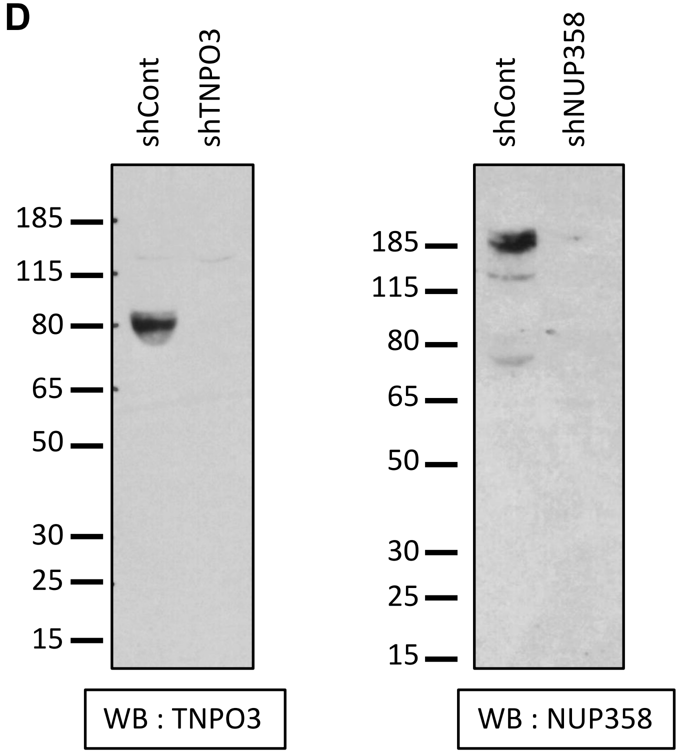

Supplementary Table 1 related to Figure 3: Data collection and refinement statistics

	6R8C
**Data collection**	
Space group	P6
Cell dimensions	
a, *b*, *c* (Å)	90.66, 90.66, 57.00
α, β, γ (°)	90.0, 90.0, 120.0
Resolution (Å)	78.52-1.91 (1.98-1.91)
R_{meas}	6.6 (73.0)
$CC_{1/2}$ (%)	99.8 (83.3)
$I / \sigma I$	17.7 (2.6)
Completeness (%)	99.3 (90.0)
Redundancy	6.5 (6.0)
Resolution (Å)	1.91
No. reflections	20758
R_{work} / R_{free}	0.19/0.23
No. atoms	1855
Protein	1612
Ligand/ion	64
Water	179
B-factors	
Protein	35.2
Ligand/ion	85.6
Water	42.3
R.m.s. deviations	
Bond lengths (Å)	0.02
Bond angles (°)	1.90

*Values in parentheses are for highest-resolution shell.
