# Supplementary material for: Cellular IP6 Levels Limit HIV Production while Viruses that Cannot Efficiently Package IP6 Are Attenuated for Infection and Replication
Source: Cell Rep. 2019 Dec 17;29(12):3983–3996.e4. doi: 10.1016/j.celrep.2019.11.050 (PMC6931105; doi:10.1016/j.celrep.2019.11.050)
Supplement: Document S2. Article plus Supplemental Information [file mmc2.pdf]

Cell Reports

Article

Cellular IP_6 Levels Limit HIV Production while Viruses that Cannot Efficiently Package IP_6 Are Attenuated for Infection and Replication

Graphical Abstract

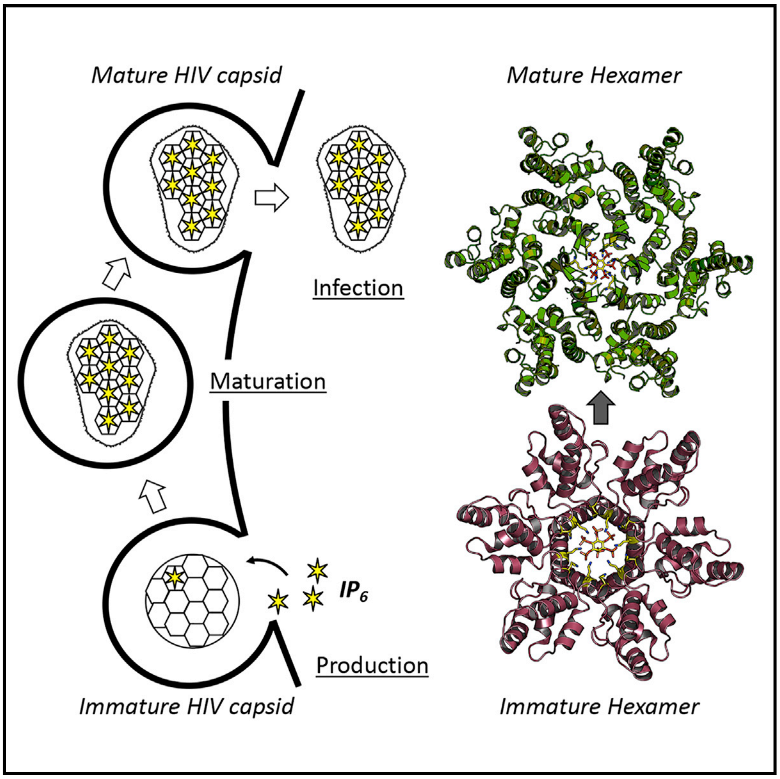

Highlights

- HIV needs cellular IP_6 to efficiently produce and assemble virions
- HIV packages IP_6 using two rings of lysine residues in immature gag hexamers
- Viruses that fail to package IP_6 do not mature properly and have unstable capsids
- Deficient IP_6 packaging prevents HIV replicating in primary cells

Authors

Donna L. Mallery, K.M. Rifat Faysal, Alex Kleinpeter, ..., Eric O. Freed, Adolfo Saiardi, Leo C. James

Correspondence

lcj@mrc-lmb.cam.ac.uk

In Brief

Mallery et al. demonstrate that HIV is crucially dependent upon the host metabolite IP_6 to produce infectious virions. Cells deficient in IP_6 produce fewer virions, while virions that fail to package sufficient numbers of IP_6 molecules are poorly infectious and fail to replicate in primary cells.

Mallery et al., 2019, Cell Reports *29*, 3983–3996
December 17, 2019 © 2019 The Author(s).
https://doi.org/10.1016/j.celrep.2019.11.050

OPEN ACCESS
CellPress

Cellular IP_6 Levels Limit HIV Production while Viruses that Cannot Efficiently Package IP_6 Are Attenuated for Infection and Replication

Donna L. Mallery,[1] K.M. Rifat Faysal,[2,6] Alex Kleinpeter,[3,6] Miranda S.C. Wilson,[4,6] Marina Vaysburd,[1] Adam J. Fletcher,[5] Mariia Novikova,[3] Till Böcking,[2] Eric O. Freed,[3] Adolfo Saiardi,[4] and Leo C. James[1,7,*]
[1]MRC Laboratory of Molecular Biology, Francis Crick Avenue, Cambridge, CB2 0QH, UK
[2]EMBL Australia Node in Single Molecule Science and ARC Centre of Excellence in Advanced Molecular Imaging, School of Medical Sciences, UNSW Sydney, Sydney NSW, Australia
[3]Virus-Cell Interaction Section, HIV Dynamics and Replication Program, Center for Cancer Research, National Cancer Institute, Frederick, MD 21702-1201, USA
[4]MRC Laboratory for Molecular Cell Biology, University College London, London, UK
[5]MRC Protein Phosphorylation and Ubiquitylation Unit, University of Dundee, Dundee, UK
[6]These authors contributed equally
[7]Lead Contact
*Correspondence: lcj@mrc-lmb.cam.ac.uk
https://doi.org/10.1016/j.celrep.2019.11.050

SUMMARY

HIV-1 hijacks host proteins to promote infection. Here we show that HIV is also dependent upon the host metabolite inositol hexakisphosphate (IP_6) for viral production and primary cell replication. HIV-1 recruits IP_6 into virions using two lysine rings in its immature hexamers. Mutation of either ring inhibits IP_6 packaging and reduces viral production. Loss of IP_6 also results in virions with highly unstable capsids, leading to a profound loss of reverse transcription and cell infection. Replacement of one ring with a hydrophobic isoleucine core restores viral production, but IP_6 incorporation and infection remain impaired, consistent with an independent role for IP_6 in stable capsid assembly. Genetic knockout of biosynthetic kinases IPMK and IPPK reveals that cellular IP_6 availability limits the production of diverse lentiviruses, but in the absence of IP_6, HIV-1 packages IP_5 without loss of infectivity. Together, these data suggest that IP_6 is a critical cofactor for HIV-1 replication.

INTRODUCTION

The HIV-1 capsid undergoes a number of transformations during viral replication: assembly, maturation, host interaction, and uncoating. Assembly occurs at the cell membrane, where the viral protein Gag polymerizes into a hexagonal structure called the immature lattice (Bush and Vogt, 2014). Once HIV virions bud from the cell, viral protease initiates maturation by cleaving Gag into multiple fragments and disrupting the immature lattice (Lee et al., 2012). The p24 capsid fragment CA then polymerizes inside virions to form a fullerene cone structure made up of a mature hexamer lattice and 12 pentamers. During post-entry infection, the capsid recruits multiple cytosolic and nuclear pore cofactors and protects HIV as it reverses transcribes its RNA into DNA (Di Nunzio et al., 2012; Lee et al., 2010; Matreyek and Engelman, 2011; Matreyek et al., 2013; Price et al., 2012, 2014; Schaller et al., 2011). Finally, the capsid undergoes uncoating freeing the viral DNA to integrate into the host genome. The molecular mechanisms that drive assembly of the immature and mature lattices during production and that trigger capsid uncoating during infection remain unclear. Understanding these processes is complicated by the conflicting structural requirements imposed by capsid function: the capsid may need to be stable for many hours inside the cell (Holmes et al., 2015) yet undergo rapid disassembly in the right place and at the right time. Definitions of even these fundamental parameters remain controversial, and there is no consensus about exactly what uncoating means and when and where it happens.

Recently, we reported that the metabolite inositol hexakisphosphate (IP_6) binds to an electropositive pore created by R18 in CA hexamers (Jacques et al., 2016; Mallery et al., 2018). We demonstrated that IP_6 binding increases HIV-1 capsid stability from minutes to hours and promotes the accumulation of DNA inside intact structures during reverse transcription (Mallery et al., 2018; Márquez et al., 2018). Importantly, we also found that HIV-1 packages IP_6 in a Gag-dependent manner during viral production. As the mature R18 pore only forms after budding, we postulated that a similar electropositive pore in the immature hexamer provided by K158 and K227 might be similarly used to bind IP_6, and this was demonstrated in a subsequent crystal structure (Dick et al., 2018). On the basis of these results, we proposed that IP_6 might be an HIV "pocket factor" that regulates stable capsid assembly. Pocket factors are small-molecule ligands that are incorporated into picornavirus capsids and dissociate upon infection to allow uncoating (Rossmann, 1994; Rossmann et al., 2002). Here we have tested the hypothesis that by driving assembly of capsid lattices, IP_6 might promote viral production and, by mediating stable capsid maturation, promote infection. By manipulating IP_6 levels in both cells and viruses, we provide physiological evidence that IP_6 is a critical HIV cofactor for viral replication.

This is an open access article under the CC BY license (http://creativecommons.org/licenses/by/4.0/).

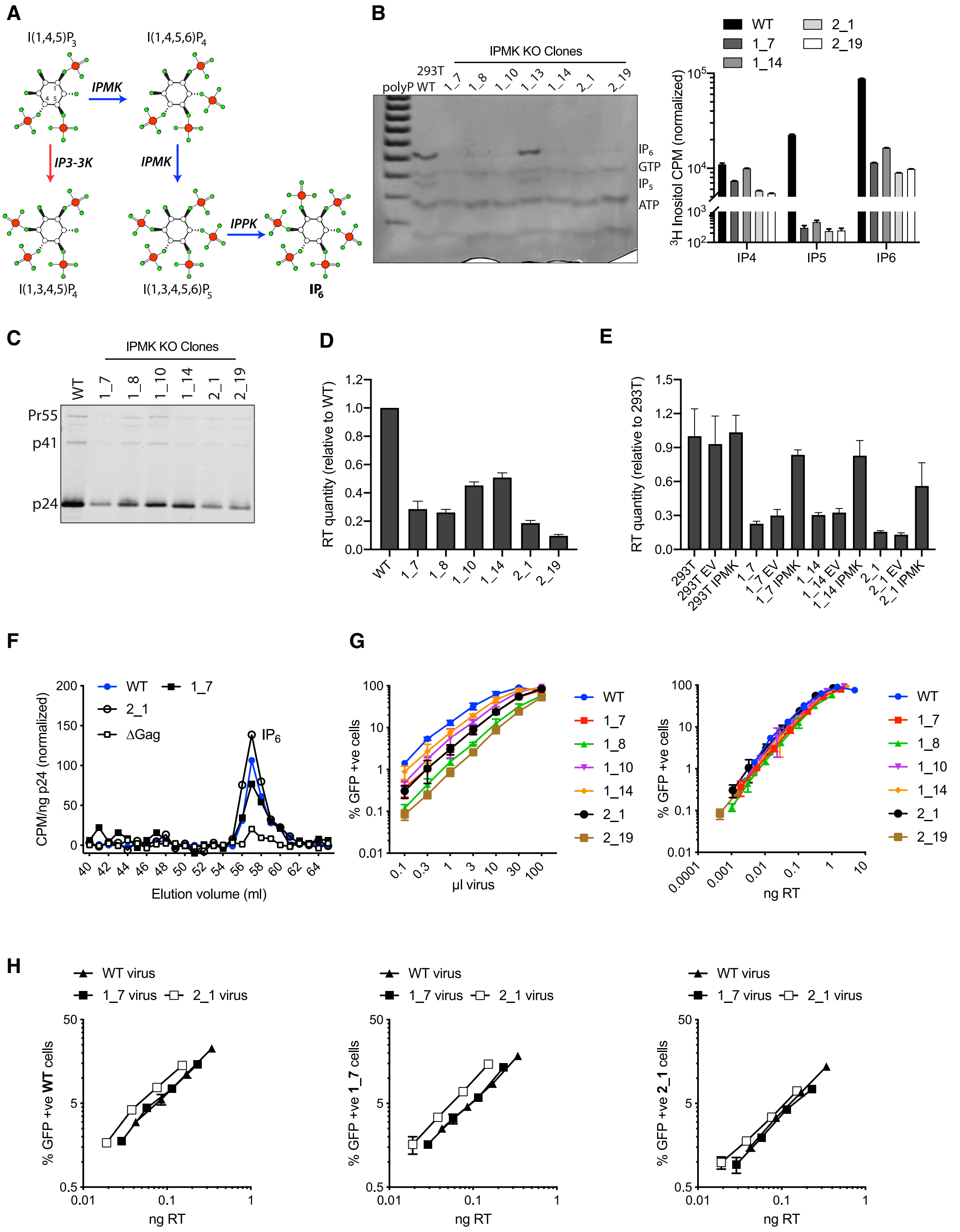

Figure 1. Depletion of IP_6 in IPMK-Knockout Cells Reduces HIV Production but Does Not Affect IP_6 Incorporation or Infectivity

(A) Biosynthetic pathway of inositol phosphates, illustrating the central role of IPMK and IPPK in IP_6 production.

(B) Analysis of IP_6 levels in IPMK CRISPR/Cas9 knockout clones by TiO_2-PAGE and toluidine blue staining of cell extracts (left), $[^3H]$inositol labeling with SAX-HPLC, and scintillation counting of fractions, normalized to background (right). Synthetic polyP was used as ladder for gel orientation.

(C) p24 western blot of pelleted virions showing p24 levels in HIV virions produced from IPMK-KO clones.

(D) Measurement of virus production through quantification of RT in viral supernatants from IPMK-KO clones. Error bars depict mean ± SD of three independent experiments. Values are represented as fold WT virus for comparison. Reduction compared with WT is statistically significant ($p < 0.0003$ in all cases).

(legend continued on next page)

RESULTS

To determine whether IP_6 is a necessary cofactor in either HIV virus production or infection, we sought to alter the availability of IP_6 in producer and target cells and determine the capability of virus to package it. IP_6 biosynthesis is a complex metabolic pathway involving the conversion of IP_3 to IP_5 by inositol polyphosphate multikinase (IPMK or IPK2), followed by phosphorylation of IP_5 to IP_6 by inositol-pentakisphosphate 2-kinase (IP5-2K, IPPK, or IPK1) (Figure 1A). As previous work has shown that genetic deletion of IPMK in mouse embryonic stem cells (ESCs) leads to a marked reduction in IP_6 levels without altering cell viability (Frederick et al., 2005), we used CRISPR/Cas9 to remove IPMK from human 293T cells (Figure S1A). Because flux through the mammalian IP pathway is incompletely understood, the effects of knocking out a particular kinase on the levels of different IP species are difficult to predict. Therefore, we directly determined the levels of IP_5, IP_6, ATP, and GTP in extracts from knockout (KO) clones using titanium dioxide purification (Wilson et al., 2015) with PAGE (TiO_2-PAGE) (Losito et al., 2009) and comparison with known standards (Figure S1B). We observed significant (~90%) IP_6 reduction in all successful IPMK knockouts, although absolute levels varied among clones (Figure 1B). IP_7 has been implicated in regulating ATP levels (Szijgyarto et al., 2011), but although ATP and GTP varied among IPMK clones, there was no consistent trend, suggesting that differences were due to clonal variation. To quantify the relative levels of inositol phosphates in IPMK-knockout clones, we grew cells in inositol-free culture media supplemented with tritiated inositol ($[^3H]$inositol) and used strong anion exchange high-performance liquid chromatography (SAX-HPLC) to isolate specific IP species. Scintillation counting of purified samples confirmed a reduction of both IP_5 and IP_6, with little change in IP_4 (Figure 1B), consistent with the availability of an alternative metabolic pathway to produce IP_4, albeit a different isomer (Figure 1A).

To test whether a reduction in cellular IP_6 levels affects viral production, we transfected a range of IPMK clones with the HIV-1 Gag-Pol expression plasmid pCRV-1 and the HIV-GFP encoding plasmid pCSGW. Analysis of Gag expression revealed that some clones had slightly higher or lower levels than parental cells (Figure S1C). In contrast, all IPMK-knockout clones produced substantially fewer viral particles, as determined by p24 blotting (Figure 1C) and quantitative ELISA for RT enzyme (Figure 1D). As with viral gene expression, the ratio of RT to p24 showed some random variability between clones but no consistent difference compared with wild-type (WT) (Figure S1D). To confirm that reduced production in the IPMK knockouts was not the result of clonal variation, we reconstituted IPMK by stable transduction (Figure S1E). Analysis of inositol phosphates in cell extracts by TiO_2-PAGE demonstrated successful restoration of IP_6 levels in IPMK-knockout clones reconstituted with IPMK but not empty vector constructs (Figure S1F). Crucially, ectopic overexpression of IPMK was sufficient to partially rescue virus production in the tested IPMK clones, without altering protein expression levels (Figure 1E; Figure S1G).

We also investigated whether depletion of IP_6 had any impact on IP_6 incorporation by virions. We purified virus from two IPMK-KO clones grown in inositol-free media supplemented with $[^3H]$ inositol as described previously (Mallery et al., 2018). The presence of IP_6 in virions was determined by scintillation counting of fractions collected using SAX chromatography. IP_6 was detected in virus produced from both tested IPMK clones, despite the reduced number of viral particles. When normalized for viral particles (CPM/ng p24), we observed no significant difference in IP_6 levels between virus produced in parental cells and IPMK knockouts (Figure 1F).

The above data suggest that cellular IP_6 levels limit the number of HIV virions that are produced but that viruses retain the same capacity for IP_6 incorporation. On the basis of this finding, we hypothesized that once normalized for viral production, HIV virions from IP_6-depleted cells should be as infectious as those produced in parental cells. Infection experiments carried out at a range of virus dilutions confirmed this prediction (Figure 1G). To test whether IP_6 levels are important in target cells, we challenged parental cells and two IPMK-knockout clones with viruses produced from each of these cell lines. Virus titration showed that all combinations were at least as infectious as parental cell-derived virus in parental cells, with 2_1 virus marginally more infectious (Figure 1H).

Assembly of an immature lattice from recombinant Gag protein can be promoted *in vitro* by both IP_5 and IP_6 (Dick et al., 2018). To test whether IP_5 can be used by HIV to promote viral production in the absence of available IP_6, we created CRISPR/Cas9 knockouts of IPPK, the enzyme responsible for conversion of IP_5 to IP_6 (Figure S2A). We used TiO_2-PAGE to show that our IPPK KOs have dramatically reduced levels of cellular IP_6 while IP_5 was unaffected (Figure 2A). Quantification of IPs following growth in $[^3H]$inositol-supplemented media further revealed that IP_5 levels in two of three IPPK clones were identical to parental cells, but IP_4 was unexpectedly increased in all clones (Figure 2B). This is in contrast to the phenotype in IPK1 yeast knockouts, in which IP_5 but not IP_4 accumulates (York et al., 1999). To determine how the different availability of IP species in IPPK knockouts alters HIV packaging and viral production, we quantified IP incorporation in virions produced from IPPK knockout clones (Figure 2C). In contrast to virus produced from IPMK KOs, IPPK viruses had little or no IP_6. However, IP_5 was now detectable in virions produced from

(E) Levels of virus production from viral supernatants collected from parental clones and the same cell lines stably transduced with either EV or IPMK. Error bars depict mean ± SD of two independent experiments. Values normalized to the levels in 293T untransduced cells.

(F) Quantification of IP_6 packaging in virions produced in wild-type cells and IPMK-KO clones by $[^3H]$inositol labeling, SAX-HPLC, and scintillation counting of fractions then normalized to background.

(G) Infectivity of viruses produced in selection of IPMK clones plotted against viral volume input (left) and plotted against quantity of RT in viral supernatants to normalize for differences in production (right).

(H) Infectivity of viruses purified from wild-type, 1_7, or 2_1 IPMK-KO producer cells and titrated onto the three different cell lines, normalized to RT levels. Error bars for infection data depict mean ± SD of three replicates from one experiment representative of three independent experiments.

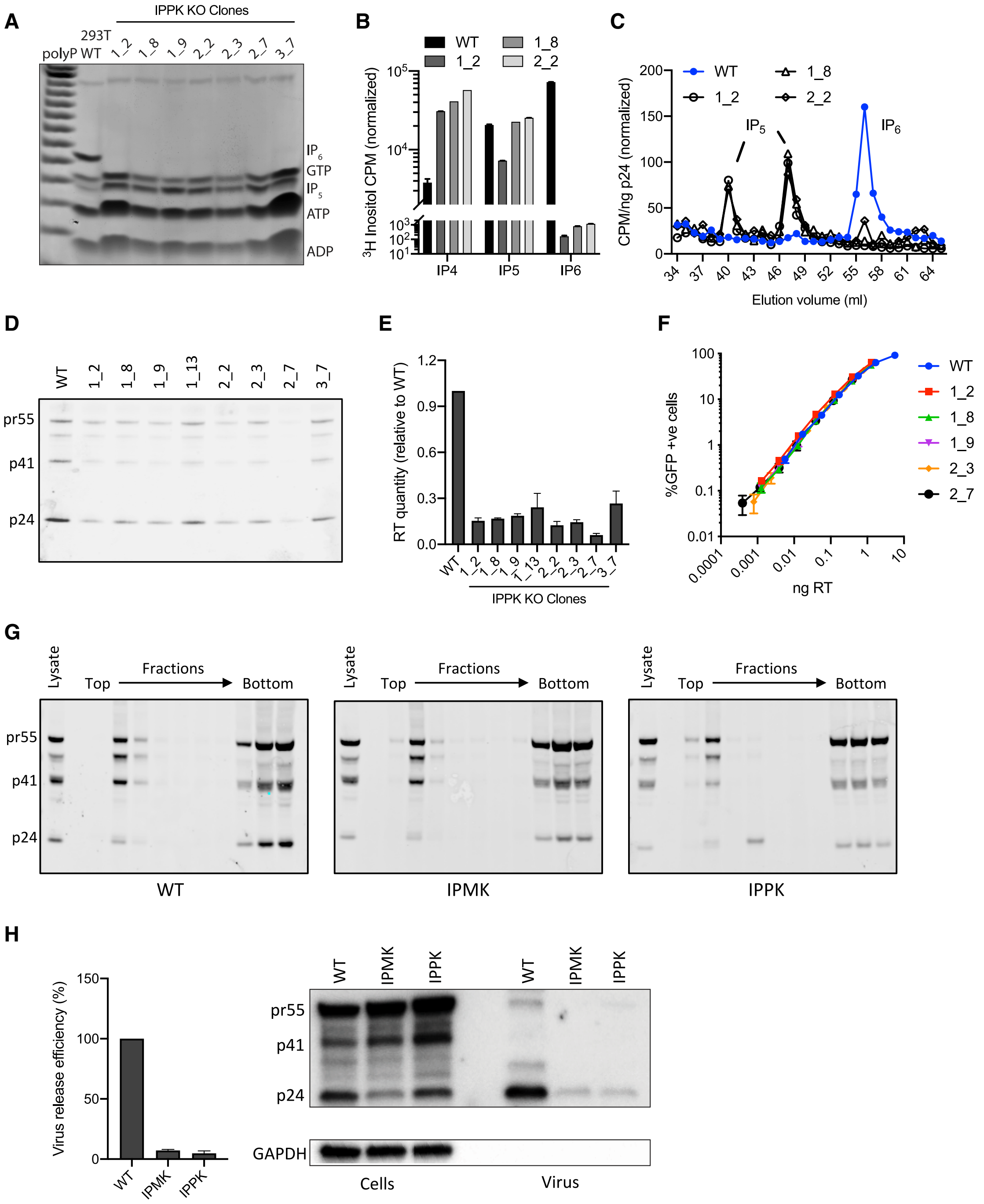

Figure 2. HIV Incorporates IP_5 in the Absence of IP_6 without Loss of Production or Infectivity

(A) TiO_2-PAGE and toluidine blue staining of cell extracts showing IP_5 and IP_6 levels in IPPK CRISPR/Cas9 knockout clones.

(B) Inositol phosphate quantification in selected IPPK-KO clones using ^3H-inositol labeling and inositol phosphate fractionation by SAX-HPLC.

(C) Quantification of IP_5 and IP_6 packaging in virions produced in wild-type and IPPK-KO cells through [^3H]inositol labeling, SAX-HPLC, and scintillation counting of fractions.

(D) p24 western blot of pelleted virions showing p24 levels in HIV virions produced from IPPK-KO clones.

(E) Measurement of virus production through quantification of RT in viral supernatants from IPMK-KO clones. Error bars depict mean ± SD of three independent experiments. Values are represented as fold WT virus, and reduction compared with WT is statistically significant ($p < 0.0012$ in all cases).

(F) Infectivity of viruses from (E), as a function of viral dose measured by RT levels. Error bars depict mean ± SD of three replicates from one experiment representative of three independent experiments.

(legend continued on next page)

all tested clones. Two discrete peaks for IP_5 were observed following SAX chromatography, which is likely the result of phosphate jumping between adjacent hydroxyl groups that occurs during IP extraction conditions (Pisani et al., 2014). Summing the two IP_5 species reveals that HIV packages a similar number of IP_5 molecules per virion when it is produced in IPPK KOs as it does IP_6 when produced in parental cells (Figure S2B). Viral protein expression in IPPK KOs was broadly similar to parental cells, except for 2_7, in which it was reduced (Figure S2C). Importantly, there was a consistent and substantial decrease in virion production in all IPPK clones as assessed both by p24 blot (Figure 2D) and RT incorporation (Figure 2E). Despite this defect in production, IPPK-derived viruses were as infectious as those from parental cells (Figure 2F), again consistent with the behavior of viruses produced from IPMK knockouts. These results suggest that HIV can substitute IP_5 for IP_6 during viral production when the latter is not available and that this does not substantially alter infectivity. The similar decrease in viral production observed in IPPK and IPMK KOs likely reflects that the IP molecule packaged by virus in these cells (IP_5 or IP_6, respectively) is at similar levels (5–10 μM).

The above data suggest that the availability of IP_5 and IP_6 in producer cells limits the production of HIV virions. This could be a result of impaired Gag recruitment to the plasma membrane or inefficient viral budding. To investigate this, we compared budding sites in WT, IPMK, and IPPK cells using a membrane flotation assay to measure Gag localization (Kutluay and Bieniasz, 2010; Ono and Freed, 1999). We observed similar levels of Gag in the membrane fractions of each cell line, suggesting that IP_6 is not involved in recruitment (Figure 2G). To determine viral release efficiency, we infected cells with VSV-G-pseudotyped env(-) pNL4-3 (pNL4-3/KFS) virus (Freed et al., 1992) and calculated the cell/virion Gag ratio. In both IPMK and IPPK KOs, there was an increase in this ratio consistent with a reduction in release efficiency (Figure 2H). These data indicate that IP_6 levels limit viral budding rather than recruitment of Gag to budding sites. Moreover, they suggest that IP_6 is limiting for viral production irrespective of whether producer cells are transfected or transduced.

To understand how HIV-1 can substitute IP_5 for IP_6 without loss of infection, we solved the crystal structure of the mature capsid hexamer in complex with the *myo* isomer of IP_5 (inositol 1,3,4,5,6-pentakisphosphate) (Table S1) and compared it with our previously solved structure with hexakisphosphate (Mallery et al., 2018). In mammals, myoIP_5 is the precursor for IP_6 synthesis by IPPK, which phosphorylates the axial 2-OH. As expected, we found that IP_5 was coordinated at the center of the HIV-1 hexamer by the ring of arginine residues at position 18 (Figures 3A and 3B). The location of the ligand at the center of the six-fold axis leads to symmetry averaging of six equivalent binding positions. However, in contrast to IP_6, which with its axial phosphate is significantly less planar, the density for IP_5 is much clearer, and the ligand can be unambiguously placed in a parallel stacking arrangement with the R18 ring (Figure 3C). This stacking conformation is reminiscent of that observed with the nonphysiological compound hexacarboxybenzene and allows multiple hydrogen bonds to form between all five equatorial phosphates and the arginine side chains in the ring (Jacques et al., 2016). The additional axial phosphate present in IP_6 would be orientated away from the R18 ring. Thus the probable reason why IP_5 can substitute for IP_6 without loss of infectivity is because interaction is driven largely by the equatorial phosphates, at least when the ligand is in a planar conformation. The packing of IP_5 above the plane of the R18 ring also places it in the cavity bounded by the β-hairpin, meaning that, like IP_6, its dissociation from the hexamer could be regulated by opening and closing of the β-hairpin (Figure 3D).

The pandemic strain HIV-1 M uses multiple protein cofactors to promote its replication in human cells. However, cofactor use is not always conserved in other diverse lentiviruses. For instance, feline immunodeficiency virus (FIV) can replicate independently of nuclear import proteins that are required by HIV-1 M (Lee et al., 2010). We therefore sought to determine whether IP_6 is an HIV-1 M specific cofactor or an evolutionary conserved component of lentiviral replication. To this end, we investigated the production of HIV-1 O, HIV-2, simian immunodeficiency virus (SIV), and FIV in cells depleted of either IPMK or IPPK (Figure S3). In all cases, we observed a reduction in the viral titer of lentiviruses produced in cells lacking either kinase (Figure 4A). This reduction in titer was accompanied by a decrease in the amount of virions produced in IPMK- or IPPK-KO cells, as assayed by RT ELISA of the supernatants (Figure 4B). The data are in agreement with our findings that reducing the availability of IP_6 in producer cells reduces viral production rather than infectivity. Moreover, there was no substantial variation in protein expression (as measured by virally encoded GFP), consistent with a requirement for IP_6 during assembly (Figure 4C). Together this suggests that IP_6 is a highly conserved lentiviral cofactor.

Next, we sought to complement our results in IP_6-depleted cells by mutating residues within Gag that are implicated in IP_6 recruitment. Previously, we hypothesized that because the R18 pore only forms after budding, IP_6 may be recruited by two lysines (K158 and K227) that form a similar charged ring in immature hexamers and bind IP_6 *in vitro* (Figure 5A). Our prediction was that mutation of these lysine residues could potentially affect both production and infection by altering recruitment of IP_6. Production might be limited because the ability of IP_6 to promote assembly of the immature lattice could be impaired, while infection might be reduced if insufficient IP_6 levels are available inside virions to promote maturation and stabilize the capsid. We mutated each lysine residue to alanine or isoleucine, with the latter residue chosen because K227I has previously been shown to be infectious (Rihn et al., 2013). As before, we measured Gag production in virus-producing cells and found

(G) Membrane flotation analysis of cell lysates from WT, IPMK-KO, and IPPK-KO cells. Western blotting of sucrose gradient fractions for Gag show that similar levels of Gag are associated with the membrane fractions. Gag precursor Pr55Gag (pr55), p41, and mature capsid protein (p24) are indicated.
(H) Virus release assays showing levels of Gag in lysates and virions after transduction of WT and KO cells with virus produced in WT cells. Graph shows relative quantification of p24 from two independent experiments and western blots. Representative data from at least two independent experiments are shown, unless otherwise indicated. CPM data are normalized to background.

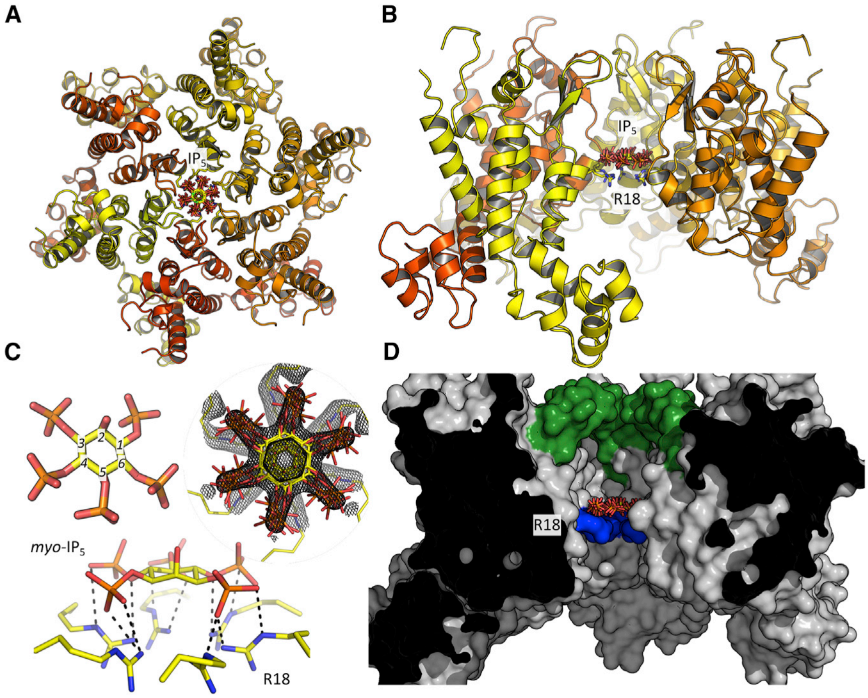

Figure 3. Structure of the HIV-1 Capsid Hexamer:IP_5 Complex

(A and B) Secondary structure representation of the complex shown from above (outside-facing, A) and side (within the capsid lattice, B). The six equivalent binding positions for IP_5 are shown, together with the location of the R18 ring.

(C) The structure of IP_5 alone and interacting with the six arginine residues at position 18. Electron density ($2F_o - F_c$) centered around the ligand is shown as a mesh contoured at 1.4σ. Putative hydrogen bond interactions between IP_5 and R18 side chains are shown as black dashes.

(D) Molecular surface of the capsid hexamer sliced through the middle to reveal the internal cavity where IP_5 is bound. All surface residues are colored gray except R18 (blue), and the β-hairpin (residues 1–12) that forms one end of the cavity (green). IP_5, positioned above the R18 ring, is also shown.

that Pr55Gag protein expression was at least as abundant as wild-type (Figure S4A). In contrast, all mutants except K227I had reduced viral production as determined both by p24 blotting and RT enzyme quantification (Figures 5B and 5C), with K227I being produced at wild-type levels by both measures.

To test whether the defects in production associated with the lysine mutants were accompanied by a loss of IP_6 recruitment, we measured IP_6 incorporation as described above. We were able to obtain data for both K227 mutants but not K158A, because too few virions were recovered during post-production processing for incorporation to be measured. Given that similar levels of K158A and K227A virions are initially produced, this suggests that K158A may be less stable. We observed a substantial reduction in IP_6 packaging per virion (Figure 5D), suggesting that the K227 ring in the immature Gag lattice is involved in recruiting IP_6 from producer cells. The finding that K227A/I mutation reduces IP_6 incorporation allowed us to investigate whether IP_6 is important for HIV infectivity in addition to its effect on production. Challenging cells with each mutant virus normalized per nanogram RT revealed that removal of either K158 or K227 results in a profound loss of infectivity (Figure 5E). Mutation of K227 reduced infection by up to 10-fold, while K158 mutants were ~100-fold less infectious than wild-type. The fact that K227A and K227I caused the same reduction in infectivity, despite the fact that only K227A is defective for production, suggests that mutation at this position affects both processes independently. Meanwhile, the finding that both mutants display similarly reduced infectivity correlates with their similarly impaired levels of incorporated IP_6. Despite having reduced IP_6 incorporation, production of K227I remained sensitive to cellular IP_6 levels and a similar reduction in production was observed in IPMK- and IPPK-KO cells as with wild-type virus (Figure S4B). Just as with wild-type virus, this production defect was not due to large changes in viral gene expression (Figure S4C). Also consistent with wild-type data, knockout of IPMK or IPPK in target cells did not alter K227I infectivity (Figure 5F).

A defect in IP6 packaging could affect the assembly and function of the mature capsid, such as supporting encapsidated reverse transcription inside the cell. Consistent with this hypothesis, all lysine mutants produced significantly less viral DNA by 4 h post-infection (Figure 5G). Importantly, within each lysine pair the same magnitude of defect was observed, with K158 mutants giving a more profound reduction in reverse transcription than K227. Taken together, this suggests that a failure in DNA synthesis is behind the reduced infectivity of the lysine mutants. The more profound impact of mutating K158 versus K227 also agrees well with the relative importance of these residues in binding IP_6 in the immature hexamer structure: K158 is primarily responsible for binding the five equatorial phosphates, whereas K227 is oriented more toward the single axial phosphate (Dick et al., 2018). Finally, we tested whether defective DNA synthesis, and consequently infection, was a kinetic effect by following infection of K227I for 150 h. The fraction of infected cells reached a plateau that remains lower for the mutant than the WT, suggesting that K227I is not merely slower in its infection kinetics (Figure 5H).

Capsid functions required for infection can be affected at the level of the virus population if maturation becomes less efficient and fewer mature capsids form or if the functional properties of all capsids are compromised. To investigate this question, we first determined whether lysine mutant viruses such as K227I can form capsids that function like wild-type and retain the ability to interact with capsid cofactors inside the cell. Nuclear pore protein Nup358 and the karyopherin TNPO3 have both been implicated as HIV cofactors whose depletion by short hairpin RNA (shRNA) reduces viral infection (Schaller et al., 2011). Nup358 binds directly to the

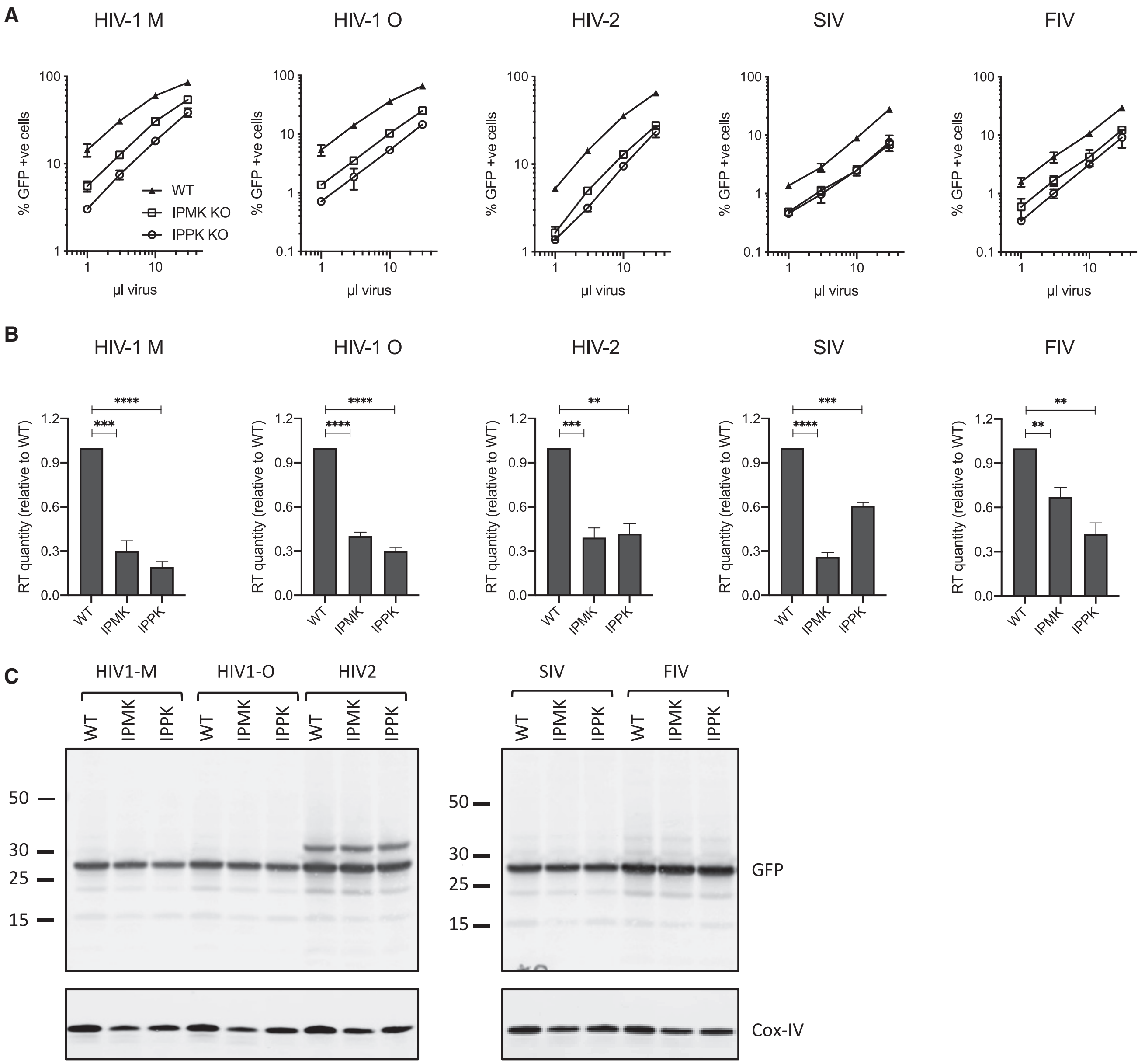

Figure 4. The Requirement for IP_6 during Viral Production Is Conserved in Diverse Lentiviruses

(A) Titers of viral supernatants for lentiviruses produced in WT, IPMK-KO, and IPPK-KO 293T cells. Titers are determined by percentage GFP infection on WT 293T cells.

(B) Quantification of viral production as determined by RT levels in viral supernatants. Values shown are mean ± SD from three independent experiments. Statistically fewer virions are produced in kinase-knockout cells ($p < 0.002$ in all cases).

(C) Western blots for GFP expression during viral production show similar protein levels for each cell line. Bottom panel shows loading control Cox-IV.

capsid via interaction with the CypA binding loop, while TNPO3 binds indirectly via cargo protein CPSF6 that binds between two monomers of the mature hexamer (Price et al., 2012, 2014). Depletion of both Nup358 and TNPO3 by shRNA reduced infection of K227I to a similar degree as wild-type virus (Figure 5I; Figure S4D). Nuclear pore protein Nup153 has also been identified as an important HIV cofactor (Matreyek and Engelman, 2011). Fusion of an FG-repeat component of Nup153 to TRIM5 generates a synthetic restriction factor TRIM-Nup153, which potently inhibits HIV infection (Matreyek et al., 2013). Importantly, Nup153 can only bind intact mature hexamers, meaning that its binding is sensitive to capsid oligomerization (Price et al., 2014). Infecting cells expressing TRIM-Nup153 revealed that mutant K227I is as sensitive to restriction as wild-type virus (Figure 5J). Finally, we tested whether K227I virus remained sensitive to the anti-capsid drug PF74. PF74 has bimodal inhibition kinetics, in which at low

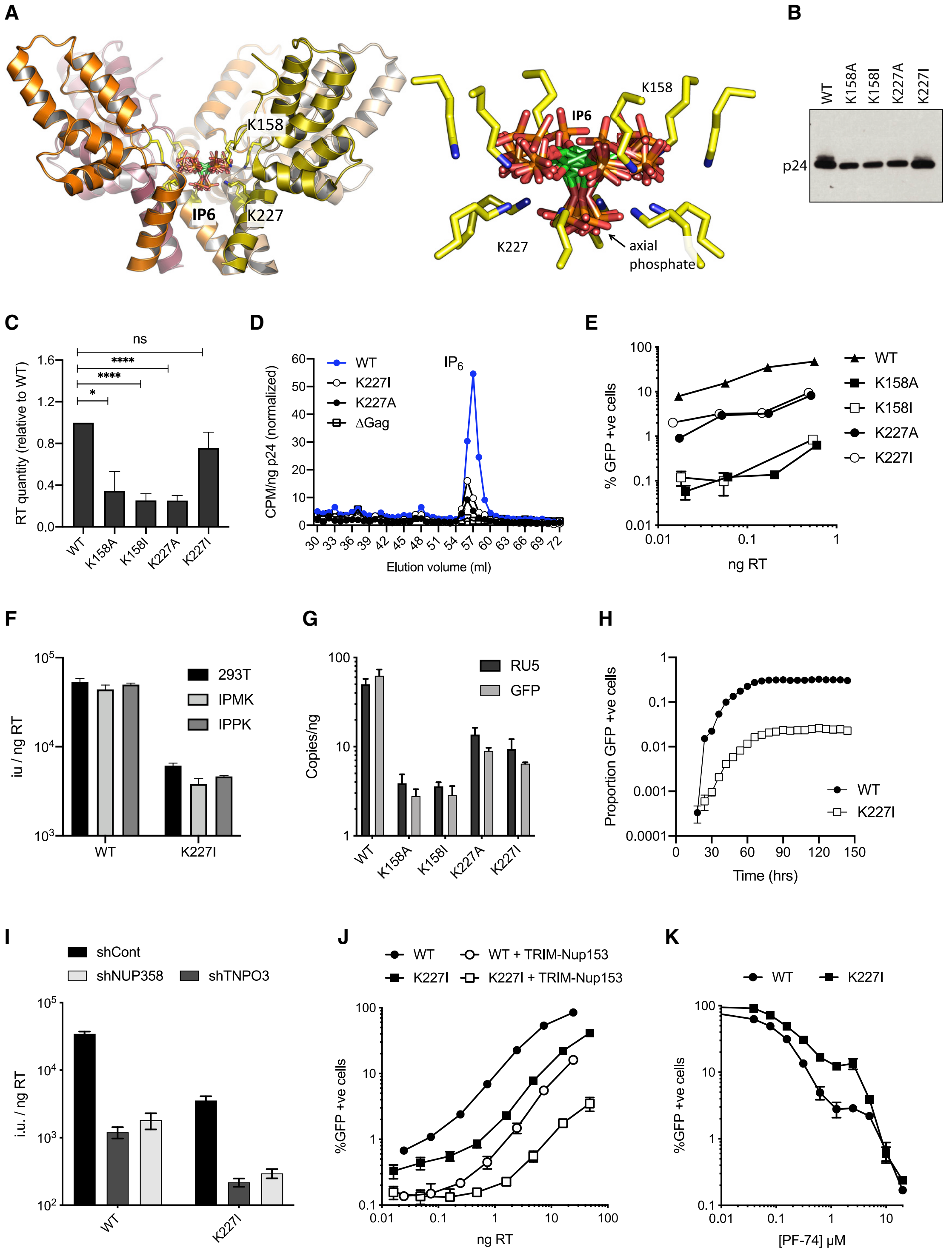

Figure 5. Mutation of K158 and K227 Rings in Immature Gag Hexamers Affects Viral Production and Infectivity

(A) View of five subunits of the immature hexamer (on the basis of PDB: 6BHR) showing the lysine side-chains responsible for coordinating IP_6 (blue sphere denotes the ε-amino group). Symmetrically equivalent molecules of IP_6 are shown with the carbon rings in green.

(B) Western blot of pelleted virions to show p24 levels in HIV wild-type and mutant virions.

(C) Quantification of mutant virus production 293T cells as determined by RT levels in viral supernatants. Error bars depict mean ± SD of three independent experiments. Values are expressed as fold change from levels of RT produced in WT virus. The reduction in virus production between WT and K158A, K158I, and K227A is statistically significant ($p = 0.0121$, $p < 0.0001$, and $p < 0.0001$, respectively).

(legend continued on next page)

concentrations it reduces infection by competing with cofactors Nup153 and CPSF6 while at high concentrations it causes irreversible and catastrophic uncoating that prevents reverse transcription (Price et al., 2014). Mutant K227I remained sensitive to PF74 and displayed similar bimodal inhibition kinetics as wild-type (Figure 5K). Interestingly, we did observe some resistance of K227I to intermediate drug concentrations. This is reminiscent of the partial rescue to PF74 inhibition in cells treated with CypA ligand cyclosporin or depleted of CPSF6 (Saito et al., 2016). These effects are thought to be due to viruses' becoming less dependent upon nuclear entry by uncoating earlier in the cytoplasm. A similar interpretation when applied to the K227I PF74 inhibition data suggests that a lack of IP_6 may reduce capsid stability. Taken together however, the data collected on capsid sensors suggest that K227I can form infectious particles that appear to be phenotypically normal with respect to host factor dependence and sensitivity to capsid-targeting inhibitors.

The above experiments do not reveal the basis for the infectivity impairment in K227I. We decided to investigate whether this may be due to K227I forming fewer stable virions. We performed single-molecule total internal reflection fluorescence (TIRF) imaging assays to compare the capsid stability of wild-type and K227I mutant virions (Figure 6A) using our CypA paint method (Márquez et al., 2018, 2019). Virions were immobilized onto coverslips attached to microfluidic flow cells and permeabilized using the membrane pore-forming protein perfringolysin O (PFO) (Figure 6A, step 1). Upon permeabilization, capsids were visualized using fluorescently labeled CypA, whereby the signal of bound CypA is constant while the capsid remains intact (Figure 6A, step 2) and then decays when the capsid uncoats (Figure 6A, step 3). Capsid stability was determined by measuring the fluorescence lifetimes of hundreds of individual virions. Example traces of individual wild-type and K227I virions are shown in Figure 6B. Collation of the data into survival curves reveals two phases, "fast" and "slow," corresponding to capsids with low and high intrinsic stability (half-lives of <1 min and 5–8 min, respectively) (Figure 6C). Importantly, K227I virions have a higher proportion of highly unstable capsids. This observation suggests that successful K227I maturation occurs less frequently than for wild-type virions, potentially explaining why the mutant has substantially reduced infectivity even though the number of produced virions is the same. Moreover, the proportion of unstable K227I capsids is likely to be even higher than measured, because the CypA-paint does not detect capsids that collapse immediately upon membrane permeabilization.

Repeating our TIRF experiments in the presence of added IP_6 revealed that the population of unstable capsids could not be stabilized even at 100 μM, providing further support for this hypothesis (Figure 6C). Importantly, the remaining K227I capsids could be stabilized by IP_6 to the same degree as wild-type, resulting in a dramatically increased lifetime. This suggests that infection by K227 is due to a fraction of properly formed and stabilizable capsids, also likely to be those that are sensitive to the capsid cofactors used above. To further correlate the stability of K227I virions on the single-molecule level with their infectivity, we carried out endogenous reverse transcription (ERT) experiments on purified capsid cores. In ERT assays, the level of DNA synthesis that occurs within intact and stable capsids can be measured by adding nuclease to degrade any exposed DNA. These assays use capsid cores extracted and purified from virions, and we typically obtained fewer K227I cores than wild-type, consistent with the fact that the mutant forms fewer stable capsids (Figure 6D). However, upon titration of nucleotides into successfully isolated cores, we observed a similar increase in encapsided DNA synthesis for both viruses (Figure 6E). Moreover, the accumulation of DNA in both wild-type and K227I capsids could be similarly promoted by the addition of IP_6. Addition of IP_5 also promoted the accumulation of DNA, consistent with the infection data of viruses produced in IPPK-KO cells, which incorporate IP_5 instead of IP_6. Taken together, the data suggest that K227I can form stable mature capsids, but far less efficiently than wild-type. This is consistent with its decreased incorporation of IP_6 and is the likely explanation of its reduced infectivity.

The above experiments were carried out in cell lines in which a single infectious cycle was measured. To investigate the importance of IP_6-recruiting residues K158 and K227 in the context of replicating virus in relevant primary cells, we performed experiments in peripheral blood mononuclear cells (PBMCs) from three independent donors. The replication of wild-type virus and mutants K158A, K158I, K227A, and K227I was determined by quantifying viral production over 20 days. All mutants displayed a profound loss of replication, with only K227I giving measurable production and then only in two of three donors

(D) Quantification of IP_6 packaging in mutants K227A and K227I after normalization for background and input virus (per nanogram p24). Both K227 mutants package similarly reduced levels of IP_6 with respect to wild-type virus. Representative data from two independent experiments are shown.

(E) Infectivity of lysine mutant viruses normalized to nanogram RT input. Each mutant pair gives similarly reduced levels of infection, with K158A/I largely impaired. Error bars depict mean ± SD of three replicates from one experiment representative of three independent experiments.

(F) Infectious titer of WT and K227I mutant viruses produced in WT 293T, IPMK-KO, and IPPK-KO cells. Infectivity is expressed as infection units (IU) per quantity of input virus (nanogram RT).

(G) Levels of reverse transcription products strong-stop (RU5) and post-strand-transfer (GFP) 4 h post-infection. Error bars depict mean ± SD of three replicates from one experiment representative of three independent experiments.

(H) Infectivity of WT and K227I virus matched for RT input over 150 h to determine whether K227I could recover infectivity over time. Infectivity is measured using an IncuCyte and determined as proportion of cell area that is GFP positive.

(I) HeLa cells stably expressing shRNA control or shNUP358 or shTNPO3 were infected with wild-type or K227I virus. Infection is quantified as infection units (IU) per quantity of input virus (nanogram RT).

(J) WT or TRIM-Nup153 expressing HeLa cells were infected with wild-type or K227I virus. The percentage infected cells was determined by percentage GFP positive cells for a range of input virus (quantified by nanogram RT).

(K) HeLa cells were infected with wild-type or K227I virus in the presence of anti-capsid inhibitor PF-74. Data are normalized to infection in the absence of inhibitor. Error bars depict mean ± SD of three replicates from one experiment representative of at least two independent experiments.

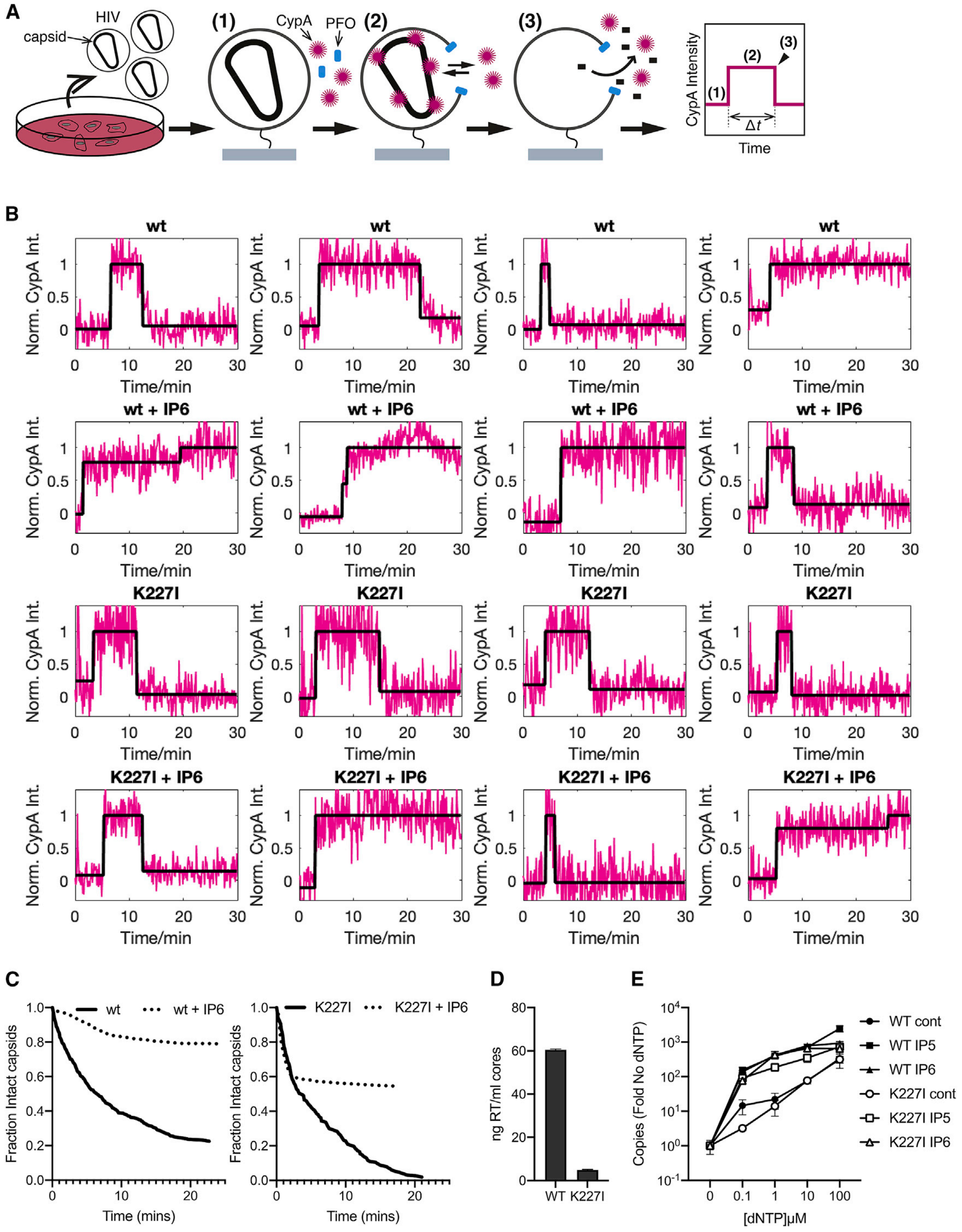

Figure 6. Addition of IP6 Is Able to Stabilize Both WT and K227I Mutant Virions

(A) Outline of single-molecule analysis for virus stability. Briefly, viral particles were immobilized, and fluorescence traces were obtained at the locations of individual virions while permeabilizing their membranes with PFO in the presence of fluorescently labeled CypA (1). CypA binds to the capsid, resulting in the appearance of a stable fluorescence signal (2). As the capsid uncoats, the CypA signal disappears (3), and the fluorescence trace returns to background levels.

(B) Example traces for individual virions demonstrating the tracking of fluorescence over time. Addition of IP6 to the virions results in a delay in the fluorescence signal reduction signaling uncoating.

(C) Capsid survival curves collated from single-virion traces showing that K227I particles have a high fraction of short-lived capsids in the presence and absence of IP_6, while the remainder of capsids is strongly stabilized by IP_6 addition for both wild-type and K227I particles.

(legend continued on next page)

and at substantially reduced levels (Figure 7). These experiments demonstrate the dependence of HIV replication in primary cells on IP_6-recruiting Gag residues.

DISCUSSION

Here we have tested the hypothesis that IP_6 is a capsid cofactor that promotes HIV-1 production and infection. Reducing cellular IP_6 levels by removal of IPMK, which converts the precursor metabolite IP_3 to IP_5, decreased viral production but did not reduce IP_6 incorporation into virions. Consistent with this, viruses produced in IP_6-depleted cells had no infectivity defect. Knockout of the downstream kinase IPPK, which converts IP_5 to IP_6, also decreased virion production without altering virion infectivity. Thus, the reduced infection previously observed using viral supernatant produced in IPPK-deficient cells (Dick et al., 2018) was probably the result of a reduction in production rather than the ability of virions to infect, as demonstrated here for both IPPK and IPMK. Our data also show that reducing cellular IP_5 and IP_6 levels affects producer but not target cells. Membrane flotation experiments in kinase KOs indicate that decreased HIV production caused by reduced cellular IP_6 levels is due to a defect in the assembly of virions at budding sites rather than recruitment of Gag to the plasma membrane. However, future studies will be necessary to investigate this process in more depth.

To complement our manipulation of IPs in producer and target cells, we also mutated the HIV Gag protein to determine how altering the ability to sequester IP_6 during assembly affects viral replication. In this second approach, we mutated two lysines in Gag, K158 and K227, that form two charged rings in the immature hexamer and bind IP_6. We hypothesized that these lysines recruit IP_6 to drive assembly of the immature lattice prior to budding and also promote stable capsid formation during maturation. In agreement with this hypothesis, we found that lysine mutation greatly reduced packaging of IP_6 into virions and significantly reduced both viral production and infection. Consistent with a role for IP_6 in assembly of the mature capsid, we also found that the infectivity loss of mutant K227I was accompanied by a dramatic reduction in the frequency of stable mature capsids.

An important difference between our cellular (IPMK and IPPK) and viral (K158 and K227) datasets is that only the latter manipulation results in reduced IP_6 incorporation per virion. This is likely because the lysine mutants have a reduced affinity for IPs and do not efficiently recruit them. Such a conclusion is supported by *in vitro* data showing that although K158 and K227 mutants form immature VLPs with near wild-type efficiency, they are unresponsive to the hugely assembly-enhancing effect of IP_6 (Dick et al., 2018). Interestingly, we observed that K227I did not have a virus production defect, in contrast to K227A. We speculate that this may be because the substitution of an isoleucine allows hydrophobic packing in the core of immature hexamers to compensate for partial occupancy and weaker binding of IP_6 within the forming lattice. The failure of isoleucine to restore production in K158I may reflect the fact that this lysine ring is dominant in IP_6 coordination and essential for IP_6 promotion of assembly. Consistent with the greater importance of K158 in IP_6 engagement, mutation of this residue gave the most profound infection and DNA synthesis defect.

The data here suggest that the previous report of abolished infectivity in K158A and K227A is actually the result of combined production and infectivity defects (Dick et al., 2018). There may be several reasons why mutation of the immature lysine rings and diminished virion incorporation of IP_6 reduces infectivity. One explanation, supported by our data, is that IP_6 is required to stabilize the mature lattice and promote capsid formation during maturation of budded virions. TIRF imaging of K227I capsids reveals that many are highly unstable and collapse with a half-life of <1 min. A reduction in the frequency with which stable K227I capsids form correlates with their reduced IP_6 packaging and explains their lower per virion infectivity. This explanation is also supported by previous *in vitro* experiments, in which IP_6 was shown to promote mature lattice assembly (Dick et al., 2018). It would nevertheless be useful to further investigate how *in vitro* virion properties predict in-cell stability and infectivity. This could be accomplished in future experiments by quantifying the structural defect associated with K227I using quantitative electron cryotomography. Interestingly, although cellular concentrations of IP_6 were sufficient to promote immature lattice assembly (10 μM), significantly higher IP_6 concentrations than those in the cytosol were required to efficiently promote formation of a mature lattice (>250 μM) (Dick et al., 2018). This is consistent with the finding that IP_6 is enriched into budded virions, creating an artificially higher IP_6 concentration during maturation (Mallery et al., 2018). This IP_6 concentration dependence may help explain why formation of the immature lattice is favored during assembly at the plasma membrane.

Although the data here support the role of IP_6 post-assembly, they do not demonstrate a requirement post-entry. That there was no infectivity loss for any virus, WT or mutant, in IPMK or IPPK cells suggests either that IPs are no longer needed, virally packaged ligands are sufficient or that viruses can obtain sufficient quantities during infection despite the reduced levels. Previously we have shown that IP_6 greatly stabilizes the mature HIV capsid after it has formed (Mallery et al., 2018). Without IP_6, capsids fall apart on a timescale (minutes) that would not support DNA synthesis or cellular infection. Our results here also show that although K227I has fewer intact capsids, those that form can be stabilized by IP_6 in both TIRF microscopy and encapsidated RT assays. These findings provide a mechanistic basis for the mutants reduced but measurable infectivity. They also support the notion that a charged polyanion is required post-entry to maintain capsid stability, albeit that this may be an abundant molecule, like ATP, obtained from the target cell. Capsid stability may be important not just to promote infection but also to evade immune sensing. A failure to incorporate sufficient IP_6 could reveal HIV to pathogen receptors in target cells by

(D) Capsid core yields determined by RT quantification (representative of at least two independent experiments).
(E) ERT assay using cores isolated from WT and K227I virions, showing a dNTP titration in the absence of IPs or in the presence of either IP_5 or IP_6. Both WT and K227I cores are competent for ERT and are stabilized by both IP_5 and IP_6 to the same extent. Data are average of three biological replicas and have been normalized to the copies of RU5 measured in the absence of dNTPs.

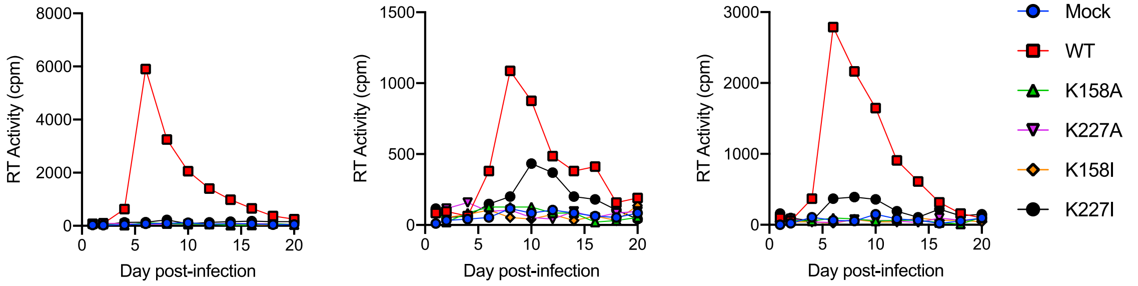

Figure 7. Capsid Mutants Prevent Efficient Replication in PBMCs

PBMCs from three donors were infected with viruses carrying capsid mutations K158A, K158I, K227A, or K227I for 2 h. Cells were cultured over 20 days and supernatants sampled every other day and assayed for RT activity.

reducing capsid stability or increasing the number of incomplete cores. For instance, this could lead to exposure of the viral genome or synthesized DNA to the STING/cGAS pathway.

A curious difference between IP_6 use in assembly versus maturation is that whereas immature hexamers use two lysine rings to engage IP_6, mature hexamers appear to use a single arginine ring. An attractive hypothesis is that the two lysine rings help preferentially recruit IP_6. The location of K227 immediately below K158 in the immature hexamer is favorable for coordinating the axial phosphate in IP_6. IP_5 lacks an axial phosphate, as it is IPPK that modifies the inositol 2-OH when it converts the molecule into IP_6 (González et al., 2010). This means that IP_5 could not make the equivalent interaction with the K227 lysine ring as IP_6, thereby thermodynamically discriminating against IP_5 binding. Nevertheless, our data show that HIV can use IP_5 when IP_6 is not available. Moreover, this substitution does not seem to drastically decrease either viral production or infection. We did observe decreased virus production in IPPK KOs, where IP_5 rather than IP_6 was specifically incorporated into virions, but this was comparable with the decrease in IPMK KOs, in which IP_6 levels were reduced to a similar level. The cellular concentration of IP_6 is normally 5-fold higher than IP_5, suggesting it is IP availability that is the primary determinant of incorporation and the reason why HIV-1 is normally highly selective for IP_6 in wild-type cells (Mallery et al., 2018).

In conclusion, our data support a model in which IP_6 is recruited from producer cells during assembly of HIV at the plasma membrane (Dick et al., 2018; Mallery et al., 2018). IP_6 is recruited by two lysines (K158 and K227) in the immature Gag hexamer, where it helps drive formation of the immature lattice. During maturation, coordination of IP_6 is disrupted when the immature lattice is cleaved by viral protease. IP_6 is then available to promote assembly of the mature capsid by coordinating the R18 pore in mature hexamers. Once inside the cell, IP_6 or another polyanion, such as ATP, may help stabilize the capsid as it transits the cytosol and undergoes DNA synthesis. Finally, and by analogy with picornavirus pocket factors, it is possible that engagement of cellular cofactors disrupts polyanion binding, allowing rapid uncoating to take place. The immature lattice lysine rings K158 and K227 and a mature capsid charged ring (e.g., R18) are highly conserved across diverse lentiviruses (Figure S5), suggesting that IP_6 is an essential component of lentiviral replication.

STAR★METHODS

Detailed methods are provided in the online version of this paper and include the following:

- KEY RESOURCES TABLE
- LEAD CONTACT AND MATERIALS AVAILABILITY
- EXPERIMENTAL MODEL AND SUBJECT DETAILS
  - Cells & Plasmids
- METHOD DETAILS
  - Infection Experiments
  - Production of [^{3}H]inositol-Labeled Virus and Cells
  - Purification and Analysis of Inositol Phosphates
  - Preparation of HIV-1 Virions
  - Virus Release
  - Replication Experiments
  - Membrane Flotation Analysis
  - CRISPR/Cas9 Knockouts
  - Western Blotting
  - Crystallization, Structure Solution, and Analysis
  - Virus Production for TIRF Microscopy
  - CypA Expression and Purification
  - TIRF Imaging of Capsids in Permeabilized Viral Particles
- QUANTIFICATION AND STATISTICAL ANALYSIS
  - Statistical Analysis
- DATA AND CODE AVAILABILITY

SUPPLEMENTAL INFORMATION

Supplemental Information can be found online at https://doi.org/10.1016/j.celrep.2019.11.050.

ACKNOWLEDGMENTS

This work was supported by the MRC (UK; U105181010), a Wellcome Trust Investigator Award (200594/Z/16/Z), a Wellcome Trust Collaborator Award (214344/A/18/Z), and the NHMRC (Australia; APP1100771 and APP1158338). A.S. was supported by the MRC (MC_UU_00012/4).

AUTHOR CONTRIBUTIONS

Conceptualization and Writing, L.C.J. and D.L.M.; Formal Analysis, D.L.M., K.M.R.F., A.K., M.S.C.W., M.V., A.J.F., T.B., E.O.F., A.S., and L.C.J.; Resources, M.N. and E.O.F; Editing, all authors.

DECLARATION OF INTERESTS

The authors declare no competing interests.

Received: April 23, 2019
Revised: October 11, 2019
Accepted: November 12, 2019
Published: December 17, 2019

REFERENCES

Azevedo, C., and Saiardi, A. (2006). Extraction and analysis of soluble inositol polyphosphates from yeast. Nat. Protoc. *1*, 2416–2422.

Böcking, T., Aguet, F., Harrison, S.C., and Kirchhausen, T. (2011). Single-molecule analysis of a molecular disassemblase reveals the mechanism of Hsc70-driven clathrin uncoating. Nat. Struct. Mol. Biol. *18*, 295–301.

Brissault, B., Kichler, A., Guis, C., Leborgne, C., Danos, O., and Cheradame, H. (2003). Synthesis of linear polyethylenimine derivatives for DNA transfection. Bioconjug. Chem. *14*, 581–587.

Bush, D.L., and Vogt, V.M. (2014). In vitro assembly of retroviruses. Annu. Rev. Virol. *1*, 561–580.

Dehairs, J., Talebi, A., Cherifi, Y., and Swinnen, J.V. (2016). CRISP-ID: decoding CRISPR mediated indels by Sanger sequencing. Sci. Rep. *6*, 28973.

Di Nunzio, F., Danckaert, A., Fricke, T., Perez, P., Fernandez, J., Perret, E., Roux, P., Shorte, S., Charneau, P., Diaz-Griffero, F., and Arhel, N.J. (2012). Human nucleoporins promote HIV-1 docking at the nuclear pore, nuclear import and integration. PLoS ONE *7*, e46037.

Dick, R.A., Zadrozny, K.K., Xu, C., Schur, F.K.M., Lyddon, T.D., Ricana, C.L., Wagner, J.M., Perilla, J.R., Ganser-Pornillos, B.K., Johnson, M.C., et al. (2018). Inositol phosphates are assembly co-factors for HIV-1. Nature *560*, 509–512.

Emsley, P., and Cowtan, K. (2004). Coot: model-building tools for molecular graphics. Acta Crystallogr. D Biol. Crystallogr. *60*, 2126–2132.

Evans, P.R., and Murshudov, G.N. (2013). How good are my data and what is the resolution? Acta Crystallogr. D Biol. Crystallogr. *69*, 1204–1214.

Frederick, J.P., Mattiske, D., Wofford, J.A., Megosh, L.C., Drake, L.Y., Chiou, S.T., Hogan, B.L., and York, J.D. (2005). An essential role for an inositol polyphosphate multikinase, Ipk2, in mouse embryogenesis and second messenger production. Proc. Natl. Acad. Sci. U S A *102*, 8454–8459.

Freed, E.O., Delwart, E.L., Buchschacher, G.L., Jr., and Panganiban, A.T. (1992). A mutation in the human immunodeficiency virus type 1 transmembrane glycoprotein gp41 dominantly interferes with fusion and infectivity. Proc. Natl. Acad. Sci. U S A *89*, 70–74.

González, B., Baños-Sanz, J.I., Villate, M., Brearley, C.A., and Sanz-Aparicio, J. (2010). Inositol 1,3,4,5,6-pentakisphosphate 2-kinase is a distant IPK member with a singular inositide binding site for axial 2-OH recognition. Proc. Natl. Acad. Sci. U S A *107*, 9608–9613.

Griffin, S.D., Allen, J.F., and Lever, A.M. (2001). The major human immunodeficiency virus type 2 (HIV-2) packaging signal is present on all HIV-2 RNA species: cotranslational RNA encapsidation and limitation of Gag protein confer specificity. J. Virol. *75*, 12058–12069.

Holmes, M., Zhang, F., and Bieniasz, P.D. (2015). Single-cell and single-cycle analysis of HIV-1 replication. PLoS Pathog. *11*, e1004961.

Ikeda, Y., Ylinen, L.M., Kahar-Bador, M., and Towers, G.J. (2004). Influence of gag on human immunodeficiency virus type 1 species-specific tropism. J. Virol. *78*, 11816–11822.

Jacques, D.A., McEwan, W.A., Hilditch, L., Price, A.J., Towers, G.J., and James, L.C. (2016). HIV-1 uses dynamic capsid pores to import nucleotides and fuel encapsidated DNA synthesis. Nature *536*, 349–353.

Kiernan, R.E., Ono, A., Englund, G., and Freed, E.O. (1998). Role of matrix in an early postentry step in the human immunodeficiency virus type 1 life cycle. J. Virol. *72*, 4116–4126.

Kutluay, S.B., and Bieniasz, P.D. (2010). Analysis of the initiating events in HIV-1 particle assembly and genome packaging. PLoS Pathog. *6*, e1001200.

Lee, K., Ambrose, Z., Martin, T.D., Oztop, I., Mulky, A., Julias, J.G., Vandegraaff, N., Baumann, J.G., Wang, R., Yuen, W., et al. (2010). Flexible use of nuclear import pathways by HIV-1. Cell Host Microbe *7*, 221–233.

Lee, S.K., Potempa, M., and Swanstrom, R. (2012). The choreography of HIV-1 proteolytic processing and virion assembly. J. Biol. Chem. *287*, 40867–40874.

Losito, O., Szijgyarto, Z., Resnick, A.C., and Saiardi, A. (2009). Inositol pyrophosphates and their unique metabolic complexity: analysis by gel electrophoresis. PLoS ONE *4*, e5580.

Mallery, D.L., Márquez, C.L., McEwan, W.A., Dickson, C.F., Jacques, D.A., Anandapadamanaban, M., Bichel, K., Towers, G.J., Saiardi, A., Böcking, T., and James, L.C. (2018). IP6 is an HIV pocket factor that prevents capsid collapse and promotes DNA synthesis. eLife *7*, e35335.

Márquez, C.L., Lau, D., Walsh, J., Shah, V., McGuinness, C., Wong, A., Aggarwal, A., Parker, M.W., Jacques, D.A., Turville, S., and Böcking, T. (2018). Kinetics of HIV-1 capsid uncoating revealed by single-molecule analysis. eLife *7*, e34772.

Márquez, C.L., Lau, D., Walsh, J., Rifat Faysal, K., Parker, M.W., Turville, S.G., and Böcking, T. (2019). Fluorescence microscopy assay to measure HIV-1 capsid uncoating kinetics in vitro. Bio Protoc. *9*, e3297.

Matreyek, K.A., and Engelman, A. (2011). The requirement for nucleoporin NUP153 during human immunodeficiency virus type 1 infection is determined by the viral capsid. J. Virol. *85*, 7818–7827.

Matreyek, K.A., Yücel, S.S., Li, X., and Engelman, A. (2013). Nucleoporin NUP153 phenylalanine-glycine motifs engage a common binding pocket within the HIV-1 capsid protein to mediate lentiviral infectivity. PLoS Pathog. *9*, e1003693.

McCoy, A.J. (2007). Solving structures of protein complexes by molecular replacement with Phaser. Acta Crystallogr. D Biol. Crystallogr. *63*, 32–41.

Murshudov, G.N., Vagin, A.A., and Dodson, E.J. (1997). Refinement of macromolecular structures by the maximum-likelihood method. Acta Crystallogr. D Biol. Crystallogr. *53*, 240–255.

Naldini, L., Blömer, U., Gallay, P., Ory, D., Mulligan, R., Gage, F.H., Verma, I.M., and Trono, D. (1996). In vivo gene delivery and stable transduction of nondividing cells by a lentiviral vector. Science *272*, 263–267.

Ono, A., and Freed, E.O. (1999). Binding of human immunodeficiency virus type 1 Gag to membrane: role of the matrix amino terminus. J. Virol. *73*, 4136–4144.

Ono, A., Demirov, D., and Freed, E.O. (2000). Relationship between human immunodeficiency virus type 1 Gag multimerization and membrane binding. J. Virol. *74*, 5142–5150.

Pisani, F., Livermore, T., Rose, G., Chubb, J.R., Gaspari, M., and Saiardi, A. (2014). Analysis of Dictyostelium discoideum inositol pyrophosphate metabolism by gel electrophoresis. PLoS ONE *9*, e85533.

Poeschla, E.M., Wong-Staal, F., and Looney, D.J. (1998). Efficient transduction of nondividing human cells by feline immunodeficiency virus lentiviral vectors. Nat. Med. *4*, 354–357.

Price, A.J., Fletcher, A.J., Schaller, T., Elliott, T., Lee, K., KewalRamani, V.N., Chin, J.W., Towers, G.J., and James, L.C. (2012). CPSF6 defines a conserved capsid interface that modulates HIV-1 replication. PLoS Pathog. *8*, e1002896.

Price, A.J., Jacques, D.A., McEwan, W.A., Fletcher, A.J., Essig, S., Chin, J.W., Halambage, U.D., Aiken, C., and James, L.C. (2014). Host cofactors and pharmacologic ligands share an essential interface in HIV-1 capsid that is lost upon disassembly. PLoS Pathog. *10*, e1004459.

Rihn, S.J., Wilson, S.J., Loman, N.J., Alim, M., Bakker, S.E., Bhella, D., Gifford, R.J., Rixon, F.J., and Bieniasz, P.D. (2013). Extreme genetic fragility of the HIV-1 capsid. PLoS Pathog. *9*, e1003461.

Rossmann, M.G. (1994). Viral cell recognition and entry. Protein Sci. *3*, 1712–1725.

Rossmann, M.G., He, Y., and Kuhn, R.J. (2002). Picornavirus-receptor interactions. Trends Microbiol. *10*, 324–331.

Saito, A., Ferhadian, D., Sowd, G.A., Serrao, E., Shi, J., Halambage, U.D., Teng, S., Soto, J., Siddiqui, M.A., Engelman, A.N., et al. (2016). Roles of

capsid-interacting host factors in multimodal inhibition of HIV-1 by PF74. J. Virol. *90*, 5808–5823.

Schaller, T., Ocwieja, K.E., Rasaiyaah, J., Price, A.J., Brady, T.L., Roth, S.L., Hué, S., Fletcher, A.J., Lee, K., KewalRamani, V.N., et al. (2011). HIV-1 capsid-cyclophilin interactions determine nuclear import pathway, integration targeting and replication efficiency. PLoS Pathog. *7*, e1002439.

Szijgyarto, Z., Garedew, A., Azevedo, C., and Saiardi, A. (2011). Influence of inositol pyrophosphates on cellular energy dynamics. Science *334*, 802–805.

Toohey, K., Wehrly, K., Nishio, J., Perryman, S., and Chesebro, B. (1995). Human immunodeficiency virus envelope V1 and V2 regions influence replication efficiency in macrophages by affecting virus spread. Virology *213*, 70–79.

Wehrly, K., and Chesebro, B. (1997). p24 antigen capture assay for quantification of human immunodeficiency virus using readily available inexpensive reagents. Methods *12*, 288–293.

Wilson, M.S., and Saiardi, A. (2018). Inositol phosphates purification using titanium dioxide beads. Bio. Protoc. *8*, e2959.

Wilson, M.S., Bulley, S.J., Pisani, F., Irvine, R.F., and Saiardi, A. (2015). A novel method for the purification of inositol phosphates from biological samples reveals that no phytate is present in human plasma or urine. Open Biol. *5*, 150014.

Winn, M.D. (2003). An overview of the CCP4 project in protein crystallography: an example of a collaborative project. J. Synchrotron Radiat. *10*, 23–25.

Yee, J.K., Friedmann, T., and Burns, J.C. (1994). Generation of high-titer pseudotyped retroviral vectors with very broad host range. Methods Cell Biol. *43* (*Pt A*), 99–112.

York, J.D., Odom, A.R., Murphy, R., Ives, E.B., and Wente, S.R. (1999). A phospholipase C-dependent inositol polyphosphate kinase pathway required for efficient messenger RNA export. Science *285*, 96–100.

Zennou, V., Perez-Caballero, D., Göttlinger, H., and Bieniasz, P.D. (2004). APOBEC3G incorporation into human immunodeficiency virus type 1 particles. J. Virol. *78*, 12058–12061.

STAR★METHODS

KEY RESOURCES TABLE

REAGENT or RESOURCE	SOURCE	IDENTIFIER
Antibodies		
Anti-HIV p24	NIH AIDS Reagent Program	183-H12-5C
anti-IPMK	Genetex	NC12
anti-Nup358	Bethyl Laboratories	A301-797A-T
anti-GFP	Abcam	ab6556
anti-TNPO3	Abcam	ab109386
IPMK gRNA 1	This paper	GCGATCGAGTCCACCCCTGA
IPMK gRNA 2	This paper	GCCCGGCCACCTGATGCGAG
IPPK gRNA B1	This paper	TGAATGGGGGTACCACGGAG
IPPK gRNA B2	This paper	AGATGGACGAGAATGAATGG
IPPK gRNA B3	This paper	AGAGGGCAATAAGAGCCTGG
Chemicals, Peptides, and Recombinant Proteins		
polyP	Sigma Aldrich	S6128
Ultima-Flo AP cocktail	Perkin Elmer	LCS537
EZ-Link Sulfo-NHS-LC-LC-Biotin	Thermo Scientific	21338
Deposited Data		
Hexamer-IP5 X-ray structure	PDB Database	6R8C
Experimental Models: Cell Lines		
HEK293T	ATCC	N/A
Experimental Models: Organisms/Strains		
HEK293T	ATCC	N/A
Recombinant DNA		
pMDG2	Mallery et al., 2018	N/A
pCRV GagPol	Mallery et al., 2018	N/A
CSGW	Mallery et al., 2018	N/A
HIV-2 GFP(Griffin et al., 2001)	Griffin et al., 2001	N/A
HIV-1 O p8.91 MVP	Ikeda et al., 2004	N/A
SIV-eGFP(Poeschla et al., 1998)	Poeschla et al., 1998	N/A
FIV FP93	Poeschla et al., 1998	N/A
pSIREN RetroQ	Mallery et al., 2018	N/A
Software and Algorithms		
GraphPad Prism 7	GraphPad	RRID: SCR_002798
AIMLESS	Evans and Murshudov, 2013	N/A
PHASER	McCoy, 2007	N/A
REFMAC5	Murshudov et al., 1997	N/A
MATLAB	The MathWorks, Inc	N/A
The PyMOL Molecular Graphics System	Schrödinger	N/A

LEAD CONTACT AND MATERIALS AVAILABILITY

Further information and requests for resources and reagents, which may require a completed Materials Transfer Agreement, should be directed to and will be fulfilled by the Lead Contact, Leo C James (lcj@mrc-lmb.cam.ac.uk).

EXPERIMENTAL MODEL AND SUBJECT DETAILS

Cells & Plasmids

293T CRL-3216 cells were purchased from ATCC and authenticated by the supplier. All cells are regularly tested and are mycoplasma free. Gag/Pol and GFP vectors were, respectively, for HIV-1 pCRV-1(Zennou et al., 2004) and CSGW(Naldini et al., 1996); for HIV-2, HIV-2 ROD Gag/Pol and HIV-2 GFP(Griffin et al., 2001); for HIV-1 O p8.91 MVP(Ikeda et al., 2004); for SIVmac, SIV3^{+} and SIV-eGFP(Poeschla et al., 1998) and for FIV FP93(Poeschla et al., 1998). Lentiviral packaging plasmid pMDG2, which encodes VSV-G envelope, was used to pseudotype infectious virions (Addgene plasmid # 12259). Cells for depletion of TNPO3 and NUP358 were produced by expression of shRNAs as previously described(Schaller et al., 2011). HEK293T and HeLa cell lines were maintained in Dulbecco's modified Eagle's medium (DMEM) with 10% FBS, 2 mM L-glutamine, 100 U/ml penicillin, and 100 μg/ml streptomycin (GIBCO) at 37°C with 5% CO_2. PBMCs were stimulated with 2 μg/ml PHA-P for 4 days before infection, then cultured in RPMI 1640 with 10% FBS, 2mM L-glutamine, 100 U/ml penicillin, 100 μg/ml streptomycin, and 50 U/ml IL-2.

METHOD DETAILS

Infection Experiments

Infections of HeLa cells were performed in the presence of 5 mg ml−1 polybrene. GFP expressing cells were enumerated on a BD LSRII flow cytometer (BD Biosciences) 2 days post-infection after fixation of cells in 4% paraformaldehyde. Values are the mean ± standard deviation. For PF74 inhibition experiments, the compound was dissolved in DMSO or DMSO and diluted in complete DMEM supplemented with polybrene as above and added to cells shortly before infection. Infections were carried out with sufficient virus to result in 10%–30% infection. Stable TNPO3 and Nup358 depletion experiments were performed by transducing HeLa cells (1×10^5) with retroviral vectors (pSIREN RetroQ) expressing specific short hairpin RNA (shRNA). Cells were selected with 10 μg/ml puromycin and stable cell-lines used in infection experiments. For further details see Schaller et al. (2011). For TRIM-Nup153 restriction experiments, a construct containing Nup153 residues 896-1475 fused to the C terminus of the tripartite domains of TRIM5 (as described in Matreyek et al., 2013) was transduced into HeLa cells and selected with puromycin. In all cases, infection was carried out in 4×10^4 HeLa cells at a multiplicity of infection (m.o.i.) between 0.1-0.3. Where viruses were quantified for levels of RT enzyme, a colorimetric RT assay kit (Roche) was used according to manufacturer's instructions.

Production of [^{3}H]inositol-Labeled Virus and Cells

[^{3}H]Inositol labeled viruses were prepared as previously described(Mallery et al., 2018). Briefly, 1×10^6 293T cells were seeded into 2 × 10cm dishes in inositol-free DMEM and left to adhere overnight. The media was replaced with 5ml inositol-free DMEM supplemented with 5 μCi/ml [^{3}H]inositol (Perkin Elmer). After 3 days incubation, an additional 5ml inositol-free media containing 5 μCi/ml [^{3}H]inositol was added onto cells, which were then transfected with 2μg each pCRV GagPol and CSGW. For determining IP levels in cells, the same procedure was used except without transfection of viral plasmids. Cells were left for a further 3 days to produce HIV-1 viral particles. After 3 days, either the cells were pelleted for IP determination or viral supernatants were collected. Viral supernatants were topped up to 30ml and pelleted over a 5ml 20% sucrose cushion) in a SW28 rotor (Beckman) at 28,000 rpm at 4°C. Pellets were resuspended in inositol free media and pelleted as previously. After the second spin, pellets were resuspended in 1ml PBS and spun at 13,000rpm at 4°C in a bench top microfuge for 60 min. Pellets were frozen at −20°C until processing. Cells were washed with PBS, then harvested by scraping, counted, and pelleted for quantification of cellular IP_6 labeling. Pellets were frozen at −20°C until processing. For comparison of virion and purified capsid core samples, p24 levels were determined by ELISA for p24 (Perkin Elmer).

Purification and Analysis of Inositol Phosphates

Analysis of unlabeled inositol phosphates was performed following previously described protocols(Pisani et al., 2014, Wilson and Saiardi, 2018). Cells were extracted using 1M perchloric acid and inositol phosphates purified using titanium dioxide (TiO_2) beads. Extracts were subsequently separated using polyacrylamide gel electrophoresis (PAGE) and visualized using Toluidine blue staining. Inorganic polyphosphate (polyP; Sigma Aldrich S6128) was used as a ladder. The relative IP_6 levels in kinase knockout cells were compared to 293T wild-type cells.

Inositol phosphates extraction and analysis by HPLC was performed modifying a previously described protocol(Azevedo and Saiardi, 2006). Cells labeled with [^{3}H]inositol as described above were resuspended in 200 μL of extraction solution (1M Perchloric acid, 5mM EDTA) and incubated on ice for 10 min. The samples were spun out at 13,000rpm at 4°C for 5 min and the supernatant recovered. Viral or cell pellets were extracted for 10 min at 100°C using 200 μL of extraction buffer and spun out as before. Supernatants from acid extractions were neutralized to pH6-8 using 1M Potassium carbonate, 5mM EDTA (approx. 100 μl) and incubated on ice with the lids open for 1-2hrs. Samples were spun at 13,000rpm at 4°C for 5 min and supernatant containing inositol phosphates was loaded onto HPLC or stored at 4°C. Extraction of IP at low pH and high temperature can induce phosphate jumping; the movement of a phosphate group to a free hydroxyl, resulting in IP isomerization. Therefore, I(1,3,4,5,6)P5, the single IP_5 isomer detectable in IPPK KOs, becomes two IP_5 species when preparing samples of viral particles.

Inositol phosphates were resolved by strong anion exchange chromatography Sax-HPLC on a Partisphere SAX 4.6° — 125 mm column (Hichrom). The column was eluted with a gradient generated by mixing buffer A (1mM EDTA) and buffer B (1mM EDTA; 1.3 M (NH4)2HPO4, pH 4.0) as follows: 0–5 min, 0% B; 5–10 min, 0%–30% B; 10–85 min, 30%–100% B; 85–95 min, 100% B. Fractions (1 ml) were collected and analyzed by scintillation counting after adding 4 mL of Ultima-Flo AP LCS537 cocktail (Perkin Elmer).

Preparation of HIV-1 Virions

Replication deficient VSV-G pseudotyped HIV-1 virions were produced in HEK293T cells using pMDG2, pCRV GagPol and CSGW as described previously(Price et al., 2014). pNL4-3 Virus Stocks were produced in HEK293T cells by transfection with HIV-1 proviral DNA (pNL4-3) using polyethylenimine (PEI) (Brissault et al., 2003). VSV-G-pseudotyped virus stocks were generated from cells co-transfected with HIV-1 proviral DNA harboring the KFS mutation in *env* (Freed et al., 1992)) and the VSV-G expression vector pHCMV-G (Yee et al., 1994) at a DNA ratio of 10:1. Viral supernatants were filtered through a 0.45-μm membrane at 48 hours post-transfection and virus was quantified by measuring RT activity.

Virus Release

293T WT and IPMK/IPPK KO cells were transduced with VSV-G-pseudotyped HIV-1 KFS (*env*-) at 1 RT(cpm)/cell. At 48 hours post-transduction, viral supernatants were ultra-centrifuged and virions were pelleted through a 20% sucrose cushion at 4°C. Virus pellets and remaining cells were lysed and western blotted for Gag. Imaging and band quantification were performed using the Sapphire Biomolecular Imager and Azure Spot analysis software (Azure Biosystems). Virus release was calculated using the following formula.

$$\frac{virus\ p24}{virus\ p24 + cell\ p24 + cell\ Pr55}$$

Replication Experiments

Peripheral blood mononuclear cells (PBMCs) from three donors were infected for 2 hours with RT-normalized virus stocks prepared by transfection of 293T cells with PEI. Cells were washed and cultured in RPMI 1640 medium with 10% FBS with penicillin (100 U/ml) and streptomycin (100 μg/ml) in a 24-well plate. Supernatants were collected every other day and virus replication was quantified by RT activity.

Membrane Flotation Analysis

Membrane flotation analysis was based on published protocols(Kiernan et al., 1998, Kutluay and Bieniasz, 2010, Ono et al., 2000). Briefly two 10cm dishes of 293T cells were seeded and transfected as for virus production. 48hrs post-transfection cells were washed twice with ice cold buffer (10mM Tris pH7.4, 100mM NaCl, 1mM EDTA) and harvested into 1ml of the same buffer. Cells were pelleted and resuspended in 500μl Hypotonic buffer (10mM Tris pH7.4, 1mM EDTA) with protease inhibitors. Cells were lysed by passing through a 25G needle multiple times, adjusted to 150mM NaCl, 1mM MgCl2 and spun at 1000 g for 10min at 4°C for 10min to remove nuclei and intact cells. Lysates were mixed to 80% sucrose and layered with 65% and 10% sucrose and centrifuged overnight at 35K rpm. 1ml fractions were removed from gradients from the top and TCA precipitated. Pelleted protein was resuspended in LDS Sample Buffer and western blotted for Gag.

CRISPR/Cas9 Knockouts

Knockout cell lines were largely produced following the IDT protocol. Briefly, 2.5μl each of crRNA and tracrRNA at 100μM were annealed. This was then mixed with 80-100 pmol Cas9 protein and incubated at 37°C for 10 min to form the Cas9/RNP mix which was kept on ice until use. 2μl of the Cas9/RNP mix was transfected into 8×10^5 cells using the Neon Transfection system, according to manufacturer's protocol (Thermo Fisher). Cells and RNP were immediately transferred into a tube containing 1ml pre-warmed antibiotic free media and subsequently into a flask containing 4ml complete media. Cells were allowed to recover for 24-72hr until being sorted to single cells. Single cell clones were grown up and DNA extracted and sequenced to analyze for CRISPR targeting. Sequences were analyzed by CRISP-ID software(Dehairs et al., 2016) and manual decoding.

Western Blotting

Samples were run on 4%–12% Bis Tris gels and transferred onto nitrocellulose membranes using iBlot (Life Technologies) and detected by ECL or by Li-COR for quantification. Anti-HIV p24 (183-H12-5C) was obtained from the NIH AIDS Reagent Program, Division of AIDS, NIAID, NIH: Anti-HIV-1 p24 Monoclonal (183-H12-5C) (Cat# 3537) from Dr. Bruce Chesebro and Kathy Wehrly (Toohey et al., 1995, Wehrly and Chesebro, 1997), anti-IPMK (NC12) from Genetex, anti-Nup358 (A301-797A-T) from Bethyl Laboratories, and anti-GFP (ab6556), anti-TNPO3 (ab109386) both from Abcam. For virus release, samples were subjected to SDS-PAGE (4%–20%), then transferred to a polyvinylidene fluoride (PVDF) membrane (Immobilon, Millipore) via semi-dry transfer (Bio-Rad Trans-Blot Turbo). The membrane was blocked for 1 hour with 5% nonfat milk in Tris-buffered saline + 0.05% Tween 20 detergent (TBST) and incubated overnight at 4°C with anti-HIV IgG. The membrane was then washed with TBST and incubated for 2 hours with anti-human horseradish peroxidase-conjugated secondary antibody and washed again. SuperSignal West Pico PLUS (Thermo Scientific) was used to reveal protein bands.

Crystallization, Structure Solution, and Analysis

CA hexamer protein was prepared exactly as described previously(Mallery et al., 2018). Crystals were grown at 17°C by sitting-drop vapor diffusion in which 100 nL protein was mixed with 100 nL precipitant and suspended above 80 µl precipitant. The structure was obtained from 12 mg/ml hexamer mixed with 14% PEG550 MME, KSCN (0.15 M), Tris (0.1 M, pH 8.5) containing 1mM of myo-IP_5 and cryoprotected with precipitant supplemented with 20% MPD. Crystals were flash-cooled in liquid nitrogen and data collected at beamline I24 at Diamond Light Source. The datasets were processed using the CCP4 Program suite(Winn, 2003). Data were indexed and integrated with iMOSFLM and scaled and merged with AIMLESS(Evans and Murshudov, 2013). Structures were solved by molecular replacement using the model 6ES8 in PHASER(McCoy, 2007) and refined using REFMAC5(Murshudov et al., 1997). Between rounds of refinement, the model was manually checked and corrected against the corresponding electron-density maps in COOT(Emsley and Cowtan, 2004). Final figures were rendered in The PyMOL Molecular Graphics System, Version 1.5.0.4 Schrödinger, LLC. The model and data were deposited in the PDB database with code 6R8C.

Virus Production for TIRF Microscopy

Replication deficient HIV-1 virions without envelope protein were produced in HEK293T cells using pCRV-1 GagPol and CSGW, biotinylated using EZ-Link Sulfo-NHS-LC-LC-Biotin (Thermo Scientific, 21338) and purified as described (Márquez et al., 2018, 2019).

CypA Expression and Purification

CypA was expressed in *E. coli* using a pET-21 vector and purified as previously described(Márquez et al., 2018). For CypA paint, CypA was labeled with Alexa Fluor 568-C2-maleimide (AF568) and binding of conjugated CypA verified by surface plasmon resonance.

TIRF Imaging of Capsids in Permeabilized Viral Particles

TIRF microscopy was carried out following the published method of Márquez et al. (2018, 2019). Briefly, biotinylated viral particles were captured onto coverslips attached to microfluidic flow cells and imaged using a custom built TIRF microscope with an ASI-RAMM frame (Applied Scientific Instrumentation), a Nikon 100 x CFI Apochromat TIRF (1.49 NA) oil immersion objective and NicoLase laser system. Immobilized virions were treated with imaging buffer containing 200 nM PFO, to permeabilize the lipid envelope, and labeled CypA (0.5 - 1 µM), to detect the capsid. TIRF images were then acquired with a frequency of 1 frame/6 s using a 561 nm laser with a 20 ms exposure time for excitation and an Andor iXon 888 EMCCD camera for detection. Single-virion fluorescence traces were extracted from the TIRF image stacks using the JIM Immobilized Microscopy analysis package (https://github.com/lilbutsa/JIM-Immobilized-Microscopy-Suite) and further analyzed in MATLAB (The MathWorks, Inc) using software adapted from previous work(Böcking et al., 2011). Briefly, the duration of the CypA signal was extracted from fluorescence traces by step-fitting using change point analysis. Capsid stability was quantified as the time difference between acquisition of Alexa Fluor 568-CypA upon permeabilization and loss of fluorescence upon capsid uncoating.

QUANTIFICATION AND STATISTICAL ANALYSIS

Statistical Analysis

Unless otherwise indicated, statistical analyses were performed with the Student's t test using GraphPad Prism 7 software (GraphPad). The number of experiments or (biological) replicates (n) used for the statistical evaluation of each experiment is indicated in the corresponding figure legends. The data are plotted as a mean ± SD or SEM as indicated. Where the mean is calculated from technical replicates, it is representative of multiple experiments that have been repeated at least twice as described within the figure legend (Figures 1F, 2C, and 5D). Data from infection experiments are plotted as the mean ± SD of three replicates from one experiment representative of three independent experiments (Figures 1G and 1H, 2F, 4A, and 5E). Where the data are given with biological errors, this has been normalized to allow multiple independent datasets to be directly compared and is described within the figure legend (Figures 1E, 2E, and 5C). The single molecule TIRF data in Figure 6 was analyzed MATLAB and full details are described in the Methods section above.

DATA AND CODE AVAILABILITY

The published article includes an X-ray structure, which has been deposited in the PDB database with the code 6R8C. Table S1 gives the data collection and refinement statistics. A full PDB validation report is also provided.

Cell Reports, Volume *29*

Supplemental Information

Cellular IP_6 Levels Limit HIV Production

while Viruses that Cannot Efficiently Package IP_6

Are Attenuated for Infection and Replication

Donna L. Mallery, K.M. Rifat Faysal, Alex Kleinpeter, Miranda S.C. Wilson, Marina Vaysburd, Adam J. Fletcher, Mariia Novikova, Till Böcking, Eric O. Freed, Adolfo Saiardi, and Leo C. James

Supplementary Figure 1 related to Figure 1: (A) Analysis of CRISPR knockout clones. Chromatograms and alignments for sequencing of CRISPR/Cas9 knockout clones of IPMK. Indels were identified using the program outlined in Dehairs, J. *et al.*(Dehairs et al., 2016), as well as manual decoding. (B) PAGE and toluidine blue staining of standard cell extracts including standard controls to demonstrate migration of IP_5, IP_6, ATP and GTP. Standards were run in the presence of cell extract to allow equivalent migration. (C) Western blot for Gag expression levels in transfected IPMK KO clones. Gag precursor Pr55Gag (Pr55), p41 and mature capsid protein (p24) are indicated. Lower panel shows loading control COX-IV. (D) Graph showing pg RT per ng p24 in virions from viral supernatants obtained from transfection of IPMK KO clones. Error bars depict mean ± SD of three independent experiments with no statistical difference to WT. (E) Western blots showing overexpression of IPMK in CRISPR knockout clones. Clones were blotted for IPMK and lower panel shows Cox-IV loading control. (F) TiO_2-PAGE and toluidine blue staining of cell extracts from IPMK KO clones stably transduced with empty vector control (EV) or a CMV-driven IPMK gene. Synthetic polyP was used for gel orientation. Gels show knockout clones are successfully reconstituted and IP_6 levels restored. (G) Western blot for Gag expression levels in IPMK KO parental clones and cells stably transduced with either EV or IPMK. Gag precursor Pr55Gag (Pr55), p41 and mature capsid protein (p24) are indicated. Lower panel shows loading control COX-IV.

Supplementary Figure 2 related to Figure 2: (A) Analysis of CRISPR knockout clones. Chromatograms and alignments for sequencing of CRISPR/Cas9 knockout clones of IPPK. Indels were identified using the program outlined in Dehairs, J. *et al.*(Dehairs et al., 2016), as well as manual decoding. (B) Comparison of total IP_5 and IP_6 incorporation in virions from IPPK KO clones shown in Figure 2C. (C) Western blot for Gag expression levels in transfected IPPK KO clones. Gag precursor Pr55Gag (pr55), p41 and mature capsid protein (p24) are indicated. Lower panel shows loading control COX-IV.

Supplementary Figure 3 related to Figure 4: Alignment of lentiviral gag sequences. Residues are highlighted by conservation, with the mature and immature charged ring residues boxed.

Supplementary Figure 4 related to Figure 5: (A) Western blot showing Gag expression levels of lysine mutants in producer cells. Gag precursor Pr55Gag (pr55), p41 and mature capsid

protein (p24) are indicated. (B) Quantification of viral production from 293T, IPMK KO and IPPK KO cells as determined by RT levels in viral supernatants. Error bars depict mean ± SD of two independent experiments. Values are expressed as fold change from levels of RT produced in WT virus. (C) Western blot for Gag expression levels in cell lysates from WT and IPMK and IPPK KO cells during WT and K227I virus production. Gag precursor Pr55Gag (pr55), p41 and mature capsid protein (p24) are indicated. (D) Western blots of cell extracts showing cell extracts from stably transduced cells, showing depletion of TNPO3 or NUP153 by expression of short hairpin RNAs.

Supplementary Table 1 related to Figure 3: Data collection and refinement statistics. Crystallographic statistics for the HIV-1 hexamer structure complexed with IP_5.

Supplementary Figure 1 related to Figure 1

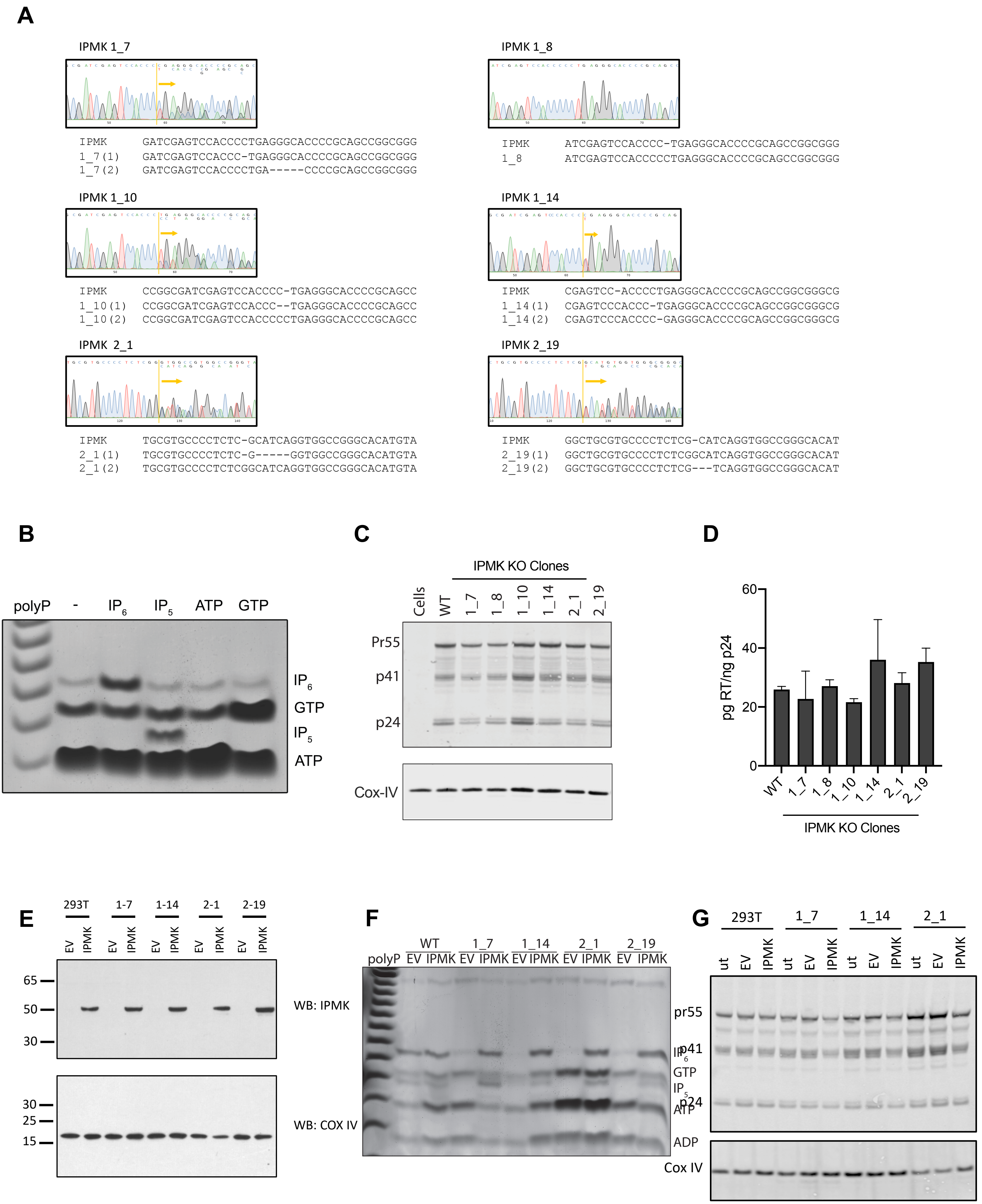

Supplementary Figure 2 related to Figure 2

A

IPPK 1_2

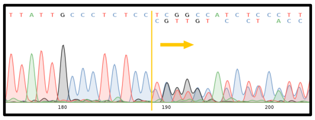

```
IPPK      TATTGCCCTCTCC-GTGGTACCCCCATTCATTCTCGTCC
1_2(1)    TATTGCCCTCTCC---------------------TCGTCC
1_2(2)    TATTGCCCTCTCCCGTGGTACCCCCATTCATTCTCGTCC
```

IPPK B1_8

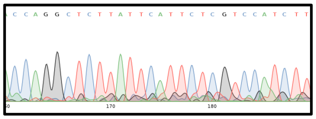

```
IPPK      GCTCTTATTGCCCTCTCCGTGGTACCCCCATTCATTCTCGTCC
1_8       GCTCTTA-----------------------TTCATTCTCGTCC
```

IPPK B1_9

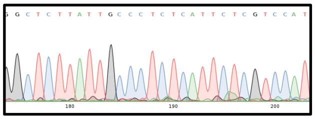

```
IPPK      ATTGCCCTCTCCGTGGTACCCCCATTCATTCTCGTCCAT
1_9       ATTGCCCTCT----------------CATTCTCGTCCAT
```

IPPK B1_13

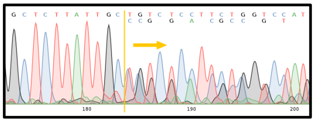

```
IPPK      GCCCTCTCCGTGGTACCCCCATTCATTCTCGTCCATCTTCCCC
1_13(1)   GCCCTCTCC----------------TTCTCGTCCATCTTCCCC
1_13(2)   GCTGGCGCA----------------------------------

IPPK      TCTTCCATGCCCAGGCGCAGCCCTGGCGCCTCGCGGGCTAGGA
1_13(1)   TCTTCCATGCCCAGGCGCAGCCCTGGCGCCTCGCGGGCTAGGA
1_13(2)   -------------------------------TCGCGGGCTAGGA
```

IPPK B2_2

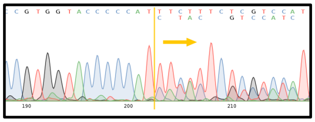

```
IPPK      CGTGGTACCCCCAT-TCATTCTCGTCCATCTTCCCCTCT
2_2(1)    CGTGGTACCCCCATTTCATTCTCGTCCATCTTCCCCTCT
2_2(2)    CGTGGTACCCCCATCT--TTCTCGTCCATCTTCCCCTCT
```

IPPK B2_3

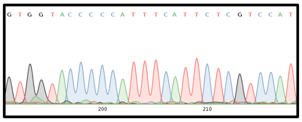

```
IPPK      CTCTCCGTGGTACCCCCA-TTCATTCTCGTCCATCTTCC
2_3       CTCTCCGTGGTACCCCCATTTCATTCTCGTCCATCTTCC
```

IPPK 2_7

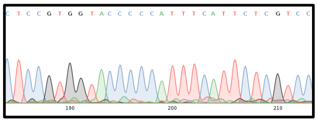

```
IPPK      GCCCTCTCCGTGGTACCCCCA-TTCATTCTCGTCCATCT
2_7       GCCCTCTCCGTGGTACCCCCATTTCATTCTCGTCCATCT
```

IPPK B3_7

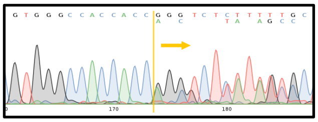

```
IPPK      CGCGTGGGCCACCACCAGG-CTCTTATTGCCCTCTCCGT
3_7(1)    CGCGTGGGCCACCACC--GGCTCTTATTGCCCTCTCCGT
3_7(2)    CGCGTGGGCCACCACCAGGTCTCTTATTGCCCTCTCCGT
```

B

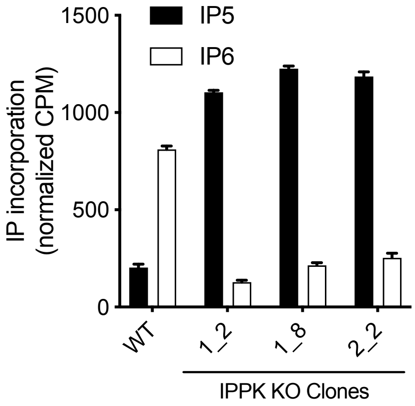

C

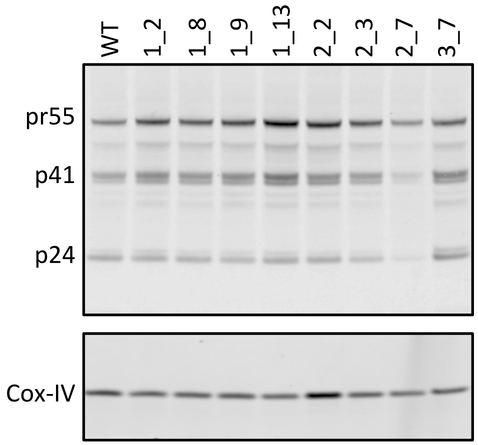

Supplementary Figure 3 related to Figure 4

```
HIV-1_M    1 MGARASVLSGGELDRWEKIRLRPGGK-KKYKLKHIVWASRELERFAVNPG--LLETSEGCRQILGQLQPSLQ--TGSEELRSLYN  80
HIV-1_O    1 MGARASVLTGSKLDAWEQIRLKPGCK-KKYRLKHLVWASRELDRFACNPE--LLETAEGNEQLLQQLEPALK--TGSESLQSLWN  80
HIV-2      1 MGARNSVLRGKKADELERIRLRPGGK-KKYRLKHIVWAANKLDRFGLAES--LLESKEGCQKILTVLDPMVP--TGSENLKSLFN  80
SIVmac     1 MGARNAVLSGKKADELEKIRLRPGGK-KKYMLKHVVWAANELDRFGLAES--LLENKEGCQKILSVLAPLVP--TGSENLKSLYN  80
FIV        1 MGNGQGRDWKMAIKRCSNVAVGVGGKSKKFGEGNFRWAIRMANVSTGREPGDIPETLDQLRLVICDLQERREKFGSSKEIDMAIV  85

HIV-1_M   81 TVATLYCVHQRIEIKDTKEALDKIEEEQNKSKKKAQQAAADTG---HSSQVSQNYPIVQNIQGQMVHQAISPRTLNAWVKVVEE- 161
HIV-1_O   81 TIAVLWCVHNRFRVEDTQQAIQKLKEVMG-SRKSAGAAKEDT----SARQTGQNYPVVANAQGQMVHQSLSPRTLNAWVKAVEE- 159
HIV-2     81 TVCVIWCIHAEEKVKDTEGAKQIVRRHLVAETGTAEKMPSTSRPTAPSSEKGGNYP-VQHVGGNYTHIPLSPRTLNAWVKLVEE- 163
SIVmac    81 TVCVIWCIHAEEKVKHTEEAKQIVQRHLVVETGTAETMPKTSRPTAPSSGRGGNYP-VQQIGGNYVHLPLSPRTLNAWVKLIEE- 163
FIV       86 TLKVFAVAGLLNMTVSTAAAAENMYSQMGLDTRPSMKEAGGK-----EEGPPQAYP-IQTVNGVPQYVALDPKMVSIFMEKARE- 163

HIV-1_M  162 KAFSPEVIPMFSALSEGATPQDLNTMLNTVGGHQAAMQMLKETINEEAAEWDRVHPVHAGPIAPGQMREPRGSD--IAGTTSTLQ 244
HIV-1_O  160 KAFNPEIIPMFMALSEGAIPYDTNTMLNAIGGHQGALQVLKEVINEEAAEWDRTHPPPIGPLPPGQIREPTGSD--IAGTTSTQQ 242
HIV-2    164 KKFGAEVVPGFQALSEGCTPYDINQMLNCVGDHQAAMQIIREIINEEAAEWDVQHP-IPGPLPAGQLREPRGSD--IAGTTSTVE 245
SIVmac   164 KKFGAEVVPGFQALSEGCTPYDINQMLNCVGDHQAAMQIIRDIINEEAADWDLQHP-QPAPQ-QGQLREPSGSD--IAGTTSSVD 244
FIV      164 GLGGEEVQLWFTAFSANLTPTDMATLIMAAPGCAADKEILDESLKQLTAEYDRTHP----PDAPRPLPYFTAAE--IMGIGLTQE 242

HIV-1_M  245 EQIGWMTN-NPPIPVGEIYKRWIILGLNKIVRMYSPTS-ILDIRQGPKEPFRDYVDRFYKTLRAEQASQEVKNWMTETLLVQNAN 327
HIV-1_O  243 EQVHWTTRPNNPIPVGDIYRKWIVLGLNKVVKMYSPVS-ILDIRQGPKEPFRDYVDRFYKTLRAEQATQEVKNWMTETLLVQNAN 326
HIV-2    246 EQIQWMFRPQNPVPVGNIYRRWIQIGLQKCVRMYNPTN-ILDIKQGPKEPFQSYVDRFYKSLRAEQTDPAVKNWMTQTLLVQNAN 329
SIVmac   245 EQIQWMYRQQNPIPVGNIYRRWIQLRLQKCVRMYNPIN-ILDVKQRPKEPFQSYVDRFYKSLRAEQTDAAVKNWMTQTLLIQNAN 328
FIV      243 QQAEARFA-----PARMQCRAWYLEALGKLAAIKAKSPRAVQLRQGAKEDYSSFIDRLFAQIDQEQNTAEVKLYLKQSLSIANAN 322

HIV-1_M  328 PDCKTILKALGPAATLEEMMTACQGVGGPGHKARVLAEAMSQVTNT----ATIMMQRGNFRNQRKMVKCFNCGKEGHTARNCRAP 408
HIV-1_O  327 PDCKQILKSLGPGATLEEMMIACQGVGGPTHKARVLAEAMAAAQDLKGGYTAVFMQRGQNPSRKGPIKCFNCGKEGHLARNCRAP 411
HIV-2    330 PDCKLVLKGLGMNPTLEEMLTACQGVGGPGQKARLMAEALKEVIGP----APIPFAAAQQ---RKAFKCWNCGKEGHSARQCRAP 407
SIVmac   329 PDCKLVLKGLGVNPTLEEMLTACQGVGGPGQKARLMAEALKEALRP----VPTPFAAAQQRGPRKPIKCWNCGKEGHSARQCRAP 409
FIV      323 ADCKKAMSHLKPESTLEEKLRACQEIGSPGYKMQLLAEALTKVQVVQ--------------SKGSGPVCFNCKKPGHLARQCREV 393

HIV-1_M  409 RKKGCWKCGKEGHQMKDCT-ERQANFLGKIWPSYKGRPGNFLQSRPEPTAPPFLQSRPEPTAPPEESFRSGVETTTPPQKQEPID 492
HIV-1_O  412 RKKGCWKCGQEGHQMKDCRNGKQANFLGKYWPPGGTRPGNYAQRQVSPSAPPMTEEMK------------GQEN----QEQKEDQ 480
HIV-2    408 RRQGCWKCGKPGHIMTNCP-DRQAGFLG--LGPWGKKPRNFPVAQVPQGLTPTAPPVDPAVDLLEKYMQQGKRQRE--QRERPYK 487
SIVmac   410 RRQRCWKCGKMDHVMAKCP-DRQAGFLG--LGPWGKKPRNFPMAQVHQGLTPTAPPEDPAVDLLKNYMQLGKQQRE--SREKPYK 489
FIV      394 KK--CNKCGKPGHLAAKCWQGNRKNSGN-----WKAGRAAPVNQMQQAVMPSAPPME---------------------------- 444

HIV-1_M  493 KELYPLTSL------------------RSLFGNDPSSQ 512
HIV-1_O  481 NELYPFASL------------------KSLFGTDQ--- 497
HIV-2    488 EVTEDLLHLEQGETPYREPPTEDLLHLNSLFGKD---- 521
SIVmac   490 EVTEDLLHL------------------NSLFGGDQ--- 506
FIV      445 ---EKLLDL----------------------------- 450
```

Supplementary Figure 4 related to Figure 5

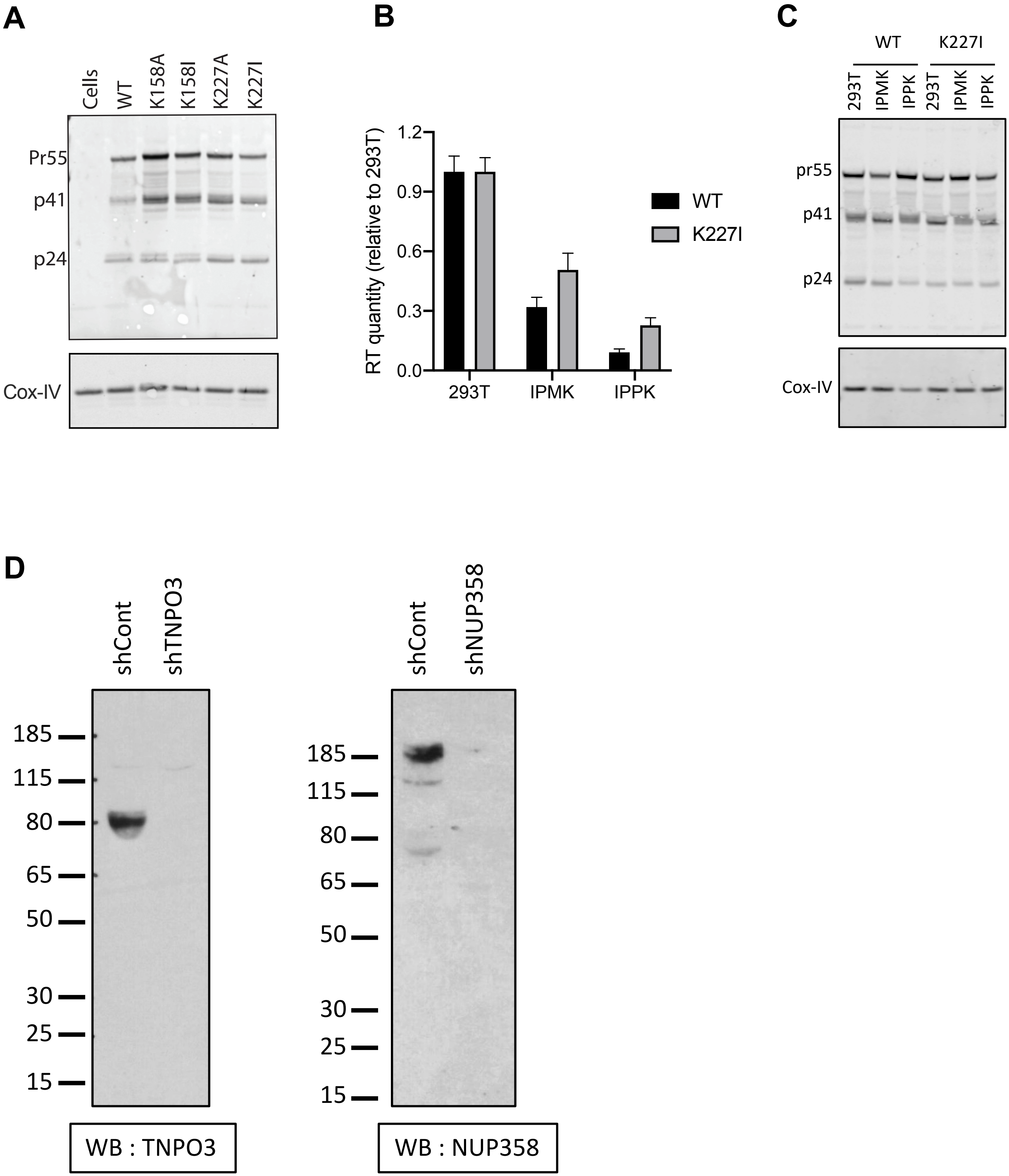

Supplementary Table 1 related to Figure 3: Data collection and refinement statistics

	6R8C
**Data collection**	
Space group	P6
Cell dimensions	
a, *b*, *c* (Å)	90.66, 90.66, 57.00
α, β, γ (°)	90.0, 90.0, 120.0
Resolution (Å)	78.52-1.91 (1.98-1.91)
R_{meas}	6.6 (73.0)
$CC_{1/2}$ (%)	99.8 (83.3)
$I / \sigma I$	17.7 (2.6)
Completeness (%)	99.3 (90.0)
Redundancy	6.5 (6.0)
Resolution (Å)	1.91
No. reflections	20758
R_{work} / R_{free}	0.19/0.23
No. atoms	1855
Protein	1612
Ligand/ion	64
Water	179
B-factors	
Protein	35.2
Ligand/ion	85.6
Water	42.3
R.m.s. deviations	
Bond lengths (Å)	0.02
Bond angles (°)	1.90

*Values in parentheses are for highest-resolution shell.
